# Ecological and human health risks of metal contamination in sediments along Egypt Western Mediterranean coast

**DOI:** 10.1038/s41598-026-39462-y

**Published:** 2026-03-11

**Authors:** Mohamed A. Hassaan, Amr G. Dardeer, Tarek O. Said, Mahmoud M. El-Mezayen, Ahmed El Nemr

**Affiliations:** 1 Environment Division, National Institute of Oceanography and Fisheries (NIOF), Kayet Bey, Elanfoushy, Alexandria, Egypt; 2 Administration of Environmental Monitoring - Ministry of Health and Population, Alexandria, Egypt

**Keywords:** Toxic elements, Health risk, Hazard quotient, Contamination, Mediterranean coast, Environmental sciences, Risk factors

## Abstract

The distribution of 24 potentially hazardous elements (PHEs) was examined in sediments gathered from several Mediterranean coastal regions in Egypt. Inductively coupled plasma-mass spectroscopy (ICP-MS) was used to analyze the samples. The lowest concentration of PHEs was recorded at 2492.95 µg/g at Salloum Station (Ia), whereas Sedi Krrir Station (X) recorded the maximum concentration at 5890.61 µg/g dry weight. The PHEs under investigation can be grouped as follows based on the highest average concentration: Ti > Fe > Al > Cu > Zn > Pb > Co > Mn > Ni > Cd > Cr. Several indicators were used to evaluate the contamination level for PHEs. Pollution indices, such as the enrichment factor (EF), geoaccumulation index (*I*_geo_), pollution load index (PLI), and contamination degree (C_d_), are determined for the recorded PHE. Principal component analysis (PCA) was employed to analyze all sediment constituents and determine their sources. To assess the degree of PHE contamination in sediments and identify the overall and specific pollution levels of different components in the sediment, PCA was utilized in conjunction with other pollution indices. According to the human risk assessment results for the PHEs found in the sediments, some of the investigated metals may pose a danger. According to the USEPA’s SQGs (sediment quality guidelines), sediments were categorized as either non-contaminated, moderately polluted, or highly polluted. Furthermore, the studied sediment samples are not contaminated with Cu, Zn, or Ni; nonetheless, C_d_ levels exceeded the thresholds of both probable effective level (PEL) and effective range medium (ERM). According to the Hazard Quotient (HQ) for cutaneous exposure route data, children’s HQ is 3–4 times that of adults.

**Supplementary Information:**

The online version contains supplementary material available at 10.1038/s41598-026-39462-y.

## 1 Introduction

Anthropogenic impacts on aquatic ecosystems affect living creatures by altering the quality of both sediment and water. The marine environment close to developed land is harmed by various sources, including pollution from industries, agriculture, and health issues^1–4^. In addition to transportation, which contaminates the environment by releasing chemicals into the atmosphere through hazardous substance leaks from containers, oil spills, ship painting, and garbage disposal^5–7^. Recent studies declared that large amounts of pollutants are released by inhabited, industrial, tourist, and commercial zones, placing the aquatic systems under excessive stress^2,4,8–10^. The quality of surface water and sediment is impacted by waste effluents released with high levels of PHEs^11–13^. Although PHEs are naturally found everywhere, the rapid rise in anthropogenic activities has led to a significant amount of metals entering aquatic habitats^14,15^.

The PHEs are the most hazardous pollutants in aquatic environments; they can exist in both suspended and dissolved forms^16,17^. Water and sediment serve as essential media for storing and transporting elements, facilitating their transformation and movement. When environmental conditions change, PHEs trapped in sediments can be released back into water; through biological, physical, and chemical processes, elements in water may absorb and/or add to deposits^18–20^. Sediments are the main reservoir for elements in the marine environment, and they contain more of these metals than seawater^21–24^. Benthic organisms feed on sediment particles, and aquatic species may uptake PHEs through their nutritional needs^25,26^. They enter the food chain and could eventually contaminate seafood, causing detrimental effects to humans^27,28^.

To preserve ecosystem health, it is crucial to evaluate PHEs in surface sediments. Sigué et al.^29^look into PHE contamination levels, ecotoxicological effects, and human health risk patterns in surface sediments in Dibang, Cameroon. ICP-MS analysis was used to examine sediment samples. The top continental crust levels were exceeded by the mean concentrations of Cd (0.27), Mn (824.75 mg/kg), Zr (224.33 mg/kg), Pb (116.41 mg/kg), and U (198.86 mg/kg). Pb and Cr (27–96 mg/kg) were similarly higher above the sediment quality limits, indicating hazardous stress reactions to benthic species in the Dibang silt. Low to high levels of sediment contamination are suggested by the contamination factor (Cf), C_d_, EF, and I_geo_. Low potential ecological and toxicity effects are indicated by potential environmental risk assessment (PERI: 40.53–161.68) and toxicity indices (TRI/TUs < 5). Each hazardous element’s non-carcinogenic risk assessment values are lower < 1 for adults and children, suggesting little health hazards. Ni, Cr, and Pb have carcinogenic risk values that are below the threshold risk level (1 × 10^–4^ and 1 × 10^–6^), indicating minimal carcinogenic health consequences. Fe, Mn, Zn, Co, V, Ni, Cr, and Zr mainly were generated from geogenic sources, whereas Cu, Cd, Sc, Pb, and U came from both geogenic and anthropogenic sources, according to results from PCA, hierarchical cluster analysis (HCA), and Pearson correlation coefficient (PCC). The consistent clustering of Cd, Pb, Cu, and U across PCA, HCA, and PCC data points to a significant anthropogenic signal associated with fertilizers, artisanal mining, household and industrial effluents, and agricultural and urban runoff. A thorough framework for evaluating and controlling sediment contamination is provided by the combination of multi-index, health risk, and multivariate approaches. These results demonstrate the critical need for focused pollution reduction, control, and environmental management initiatives in Dibang, Cameroon.

Numerous negative health consequences, such as cancer, renal damage, IQ (Intelligence Quotient) loss, behavioral problems, and, in rare instances, mortality from excessively high levels of PHEs, have been connected to them^30–32^.

The distribution, origins, and ecological hazards of potentially toxic elements (PTEs) in the marine and beach sediments of Tangier Bay, located in the south-western Mediterranean, are examined^33^. The physicochemical characteristics and metal concentrations of As, Cr, Zn, Cd, Pb, and Cu were examined in sediments from 22 different places. Beach sediments exhibited greater levels of Cd (up to 35.85 mg/kg), indicating urban runoff pollution, while marine sediments close to industrial discharge sites had elevated levels of As (up to 40.28 mg/kg), Cr (40.60 mg/kg), and Zn (57.29 mg/kg). Both natural and man-made causes have an impact on the regional variation in metal levels. Baseline concentrations are established by geological features and river inputs; however, pollution is exacerbated by industrial processes, wastewater discharge, and maritime operations. Significant pollution, particularly from Cd and As, was identified through risk assessment using geoaccumulation, enrichment factor, and ecological risk indices. These results underscore the need for targeted remediation initiatives to protect the environmental health of Tangier Bay. Simou et al.^34^aimed to assess the metal contamination and environmental hazards at the well-known Marqala Beach. For a thorough examination of the region, both wet and dry beach sediments were gathered from this coastline.

The evaluation included determining the amounts of heavy metal pollution as well as the physicochemical characterization of the sediments. The ecological and health risks were assessed using several pollution indices, including the Igeo, EF, CF, modified contamination degree (mCd), PLI, and PERI. These indices demonstrate the effects of human activity and show that increasing levels of As, Cr, Zn, and Pb are the main cause of contamination. Additionally, SiO_2_ was the most prevalent element in the sediment samples, followed by CaO and Al_2_O_3_, as determined by X-ray fluorescence (XRF) analysis. This implies a mix of mineralogical and compositional elements. In agreement with the XRF results, X-ray diffraction (XRD) analysis confirmed the dominance of quartz and the presence of calcium silicate and aluminosilicate minerals. Furthermore, the macrostructure and granulometric analyses of the scanning electron microscope (SEM) revealed a non-crystalline structure with a sandy texture, whereas the polluted sediments displayed smaller particle sizes, consisting of organic matter on quartz crystals and tiny particles (oxides).

In light of recent findings, Afahnwie et al.^35^use single and cumulative pollution indicators to assess stream sediments for metal exploration, pollution, and ecological implications. ICP-MS was used to determine the metal concentrations in stream sediments from Manjo and its surrounding areas. When compared to the upper continental crust (UCC) geochemical threshold values, the sediment samples show high concentrations of Fe, Pb, Hg, Ga, Zn, Cr, Cu, Co, Ni, V, Sn, Ce, La, U, and Th, suggesting some significant anomalies for possible base metal mineralization and exploration implications. Metallic pollution and the degree of sediment contamination are indicated by the single and cumulative pollution indices. High ecological and significant toxicity risks are indicated by the potential environmental risk index (RI: 17.682–457.791) and toxic risk index (TRI: 2.243–29.981), suggesting that metals may have biological effects on species that live in sediment. High toxicological effects and reaction stress of metals (Pb, Cu, Cr, and Ni) to aquatic ecosystem flora and fauna are suggested by sediment quality guidelines. The research area’s metallic pollution and base metal enrichment in sediments are caused by both natural and man-made input sources. The sediments were primarily deposited in a non-marine environment and come from quartzose and mafic sedimentary provenances. The sediments are also mature, exhibit moderate to severe weathering, and are primarily quartz-rich. They suggest that in order to reduce anthropogenic inputs, more attention should be paid to monitoring the point sources of metals entering aquatic environments from human-induced activities. Ensuring the ecological sustainability of the water sediments’ environment will require a dedication to research and the application of science-based tactics. Furthermore, the natural lithological contributions to the geochemistry of the sediment are noted as having significance for upcoming mineral exploration projects.

Egypt’s Mediterranean coasts are home to a variety of industrial operations, including petroleum refineries, metallurgy, food production, and cement manufacturing. Shipbuilding, retail stocking, tourist, business, commercial discharges, and urban wastewater are examples of port operations^36–38^. As demonstrated in multiple studies, marine sediments from industrialized coastal regions are heavily polluted with elements. Therefore, examining the distribution of metals in surface sediments is pertinent for evaluating pollution in marine settings^39–41^.

The quantities and spatial-temporal fluctuations of 18 PTEs in the sediments of the Felent Stream Basin were carefully investigated, which also assessed the ecotoxicological risks associated with these PTEs in both dry and wet seasons^24^. The following is a ranking of the average PTE contamination levels in sediments: Fe > Al >  Zn > Mn > Sr > Pb > Ba > Ni > Cu > As > Cr >  Li > V > Cd > Co > Sb > Se > Hg. Surprisingly, sediment samples showed a threefold average increase in PTE concentrations during the rainy season. The basin exhibits low pollution levels during the dry season and moderate pollution levels during the wet season, as indicated by ecological risk assessment indicators. In both seasons, the carcinogenic risks associated with As and the non-carcinogenic hazards for the PTEs under study remained below the threshold values. The Yoncalı District, a well-known thermal tourism destination, was identified by statistical analyses as the primary source of sediment contamination in the Felent Stream Basin [^42^]. The primary stagnant water body in the area, Enne Dam Lake, was found to be the least contaminated element. It serves as a natural filter for the basin, considerably lowering the PTE levels in the sediment.

Urban street dust contributes to environmental contamination and deterioration of air quality by acting as a sink and a secondary source of PTEs. Öncü et al.^43^used geochemical and statistical techniques to examine the concentrations, spatial distribution, ecological risks, sources, and related health risks of specific PTEs (Al, As, Cd, Co, Cr, Cu, Fe, Mn, Ni, Pb, Zn) in < 63 μm street dust samples taken from 29 locations throughout Istanbul, Turkey. ICP-MS was used to measure elemental concentrations, and Nemerow’s pollution indices (NPI), geo-accumulation (Igeo), enrichment factor (EF), contamination factor (CF), and potential ecological risk index (PERI) were used to assess contamination. Three main sources were found via Positive Matrix Factorization (PMF): natural/soil inputs (29.4%), transportation emissions (31.3%), and industrial runoff (39.4%). Ingestion was identified as the main exposure pathway in health risk evaluations. The results of the Monte Carlo simulation showed that the 95th percentiles of THI (3.57) and TCR (2.61 × 10^–2^) were higher than the suggested levels for children, indicating possible carcinogenic and non-carcinogenic concerns; however, the risks for adults were mainly within acceptable bounds. The main contributors to non-carcinogenic hazards were traffic-related elements like Pb, Cu, and Zn, with further implications for inhalation exposure through dust resuspension. Localized dust exposure was found to pose serious health hazards, even when the Air Quality Index (AQI) was below 50, indicating generally favourable atmospheric conditions during the study period. To reduce PTE exposure and improve urban environmental health, these findings underscore the need for coordinated mitigation techniques, including dust suppression, traffic emission regulations, and urban greening.

The Yeşilırmak and Kızılırmak rivers have an impact on the coastal sediments of Samsun province. Varol et al.^44^investigated the amounts, sources, ecological dangers, and health effects of metal poisoning. Using ICP-MS, surface sediment samples from 18 locations representing different land-use types were examined for 13 metals (Ba, Cr, Mn, Ni, Co, Sr, Cu, Fe, Zn, Cd, Pb, As, and Hg). The results of the spatial assessment revealed that while Cd, Pb, Cu, and Hg demonstrated anthropogenic enrichment, particularly at sites near industrial and agricultural areas, substantial concentrations of Cr and Ni were found at all locations, primarily due to natural geological formations. The levels of contamination and ecological risks related to the metals were assessed using SQG and pollution and ecological risk indices. Cr and Ni were the primary drivers of the moderate to high pollution levels indicated by the pollution indices (EF, CF, PLI, NPI, and MPI). Cd posed a moderate risk in locations impacted by industrial and agricultural activities, according to environmental risk indices (Er, RI, and NRI). Some locations exhibited hazardous risk potential due to high Ni and Cr concentrations, as indicated by SQGs-based indices (mERM-Q, TRI, and HQc). The levels of contamination and ecological risks related to the metals were assessed using SQGs and pollution and ecological risk indices. Cr and Ni were the primary drivers of the moderate to high pollution levels indicated by the pollution indices (EF, CF, PLI, NPI, and MPI). Cd posed a moderate risk in locations impacted by industrial and agricultural activities, according to ecological risk indices (Er, RI, and NRI). Some locations exhibited hazardous risk potential due to high Ni and Cr concentrations, as indicated by SQGs-based indices (mERM-Q, TRI, and HQc). According to health risk evaluations, children are more sensitive than adults, and ingestion is identified as the primary exposure channel. Three main sources of metals were determined by the Absolute Principal Component Score-Multiple Linear Regression model: anthropogenic activities (32.35%), natural weathering (21.56%), and mixed sources (46.09%). To maintain the health of coastal ecosystems and ensure public safety, the results underscore the importance of establishing local background values and sediment quality criteria, reaffirming the need for integrated management techniques.

Three critical locations along Egypt’s western Mediterranean coast serve as the study areas: Abu Qir Bay, al-Mex Bay, and Marsa Matrouh. Abu Qir Bay, a semi-circular basin, is located 35 km east of Alexandria, Egypt.

The area is affected by human activities, including several types of discharge, such as untreated sewage, industrial wastes, and agricultural runoff water. The Abu Qir Bay stations can be defined as deltaic inner-shelf biotopes associated with fertile marine habitat, which is evident from the relatively high values of fine sediments of silt and clay, and high contents of mud^45^. Al-Mex Bay is circular, with a diameter of roughly 15 km between Agami and the Western Harbor^45^. Several industrial facilities, including the Alexandria Petroleum Company (APC), Alexandria Mineral Oils Company (AMOC), and Alexandria Portland Cement Company (APCC), release effluent straight into the bay^46^. Marsa Matrouh is situated on the western Egyptian Mediterranean coast, 184 km west of Alamein. The rocky ridges of Marsa Matrouh serve as a vital habitat for many marine plant and animal species^47^. The purpose of that study was to identify which metals might have harmful ecological effects on marine life. In water, the average concentrations of Cd, Cu, Fe, Ni, Pb, and Zn were 2.381 ± 3.389, 9.307 ± 14.159, 68.969 ± 9.397, 2.642 ± 1.004, 16.712 ± 8.469, 31.168 ± 15.322 µg/l, and 0.755 ± 1.584, 3.972 ± 2.180, 15.210 ± 4.434, and 24.608 ± 7.706 µ. Except for two stations (Cleopatra and El-Obayed), which showed higher values above the allowable threshold of Cd during autumn 2010, the concentrations of the six metals under investigation in the water were within acceptable bounds. The evaluation of metal pollution in sediment and water was examined. According to the PHEs contamination index, water is not seriously contaminated by the metals under investigation. Threshold effect concentrations (TEC HQ) for sediment samples were less than 1, except for Cd, which had a value greater than 1. This suggests that Cd may have toxic adverse ecological effects on benthic organisms, whereas Cu, Ni, Pb, and Zn are expected to have rare adverse ecological effects^47^.

The Nile Delta coast stretches from Port Said eastward to Alexandria westward along the central portion of Egypt’s Mediterranean coast. To determine the degree of pollution for nine PHEs—Fe, Mn, Zn, Ni, Cu, Cr, Cd, Pb, and Ba—six brief sediment cores were taken throughout the Nile Delta region^48^. The sediment characteristics of the core samples were discovered through geochemical analysis. Nile fine-grained sand made up the majority of the sediment, with varying amounts of organic materials, carbonate, and PHEs. They primarily relate to a scale of effluent discharge exposure relevant to human influences affecting coastal ecology. According to the environmental indices used, the eastern edge of the Nile Delta, represented by the Port Said core, is categorized as a polluted area, while the western and central sites are classified as unpolluted areas. Metal contamination levels were found to vary, with high levels of Cd, significant levels of Cr, and moderate levels of Zn, Ni, Cu, Mn, and Fe. The anthropogenic effects of industries and port operations have a significant impact on the Port Said site. To prevent the further deterioration of the Nile Delta coast, it is recommended to mitigate ecological risks and halt pollution from spreading westward^48^. The results of PHEs in 20 surficial sediments taken from several points along the Egyptian Mediterranean Sea were examined^48^. They revealed that Fe had the highest mean value (243–38045 µg g^− 1^), followed by Mn (17–1086 µg g^− 1^), and lower concentrations were found for Co (0.43–26.39 µg g^− 1^) and Cd (0.04–0.47 µg g^− 1^). The risk assessment revealed that Cd posed the highest ecological risk (Er = 21.52), followed by Pb (Er = 3.01), while Zn presented the lowest risk (Er = 0.23). Both the ecotoxicological index method and the potential ecological risk index (RI) suggested that the combined ecological risk of the studied metals may be low. Multivariate statistical analysis (Cluster and Factor analysis) indicated that the lithogenic factor dominates the distribution of most of the considered metals in the study area.

The present work was aimed to (i) assess the concentrations and distribution of elements such as Li, B, Na, Mg, Al, K, Ca, Ti, Cr, Mn, Fe, Co, Ni, Cu, Zn, Ga, Se, Sr, Ag, Cd, In, Ba and Pb in Egyptian Mediterranean coast sediments, The elements selected frequently have proven functions as vital nutrients for the health of humans, animals, or plants. On the other hand, they can be known hazardous heavy metals or possible environmental pollutants (such as lead, cadmium, and chromium) whose levels need to be monitored. (ii) assess the ecological danger of these metals for potentially harmful elements such as Al, Ti, Cr, Mn, Fe, Co, Ni, Cu, Zn, Ag, Cd, and Pb by evaluating the CD and EF of PHEs in the sediment using the Igeo and PLI. (iii) perform multivariate statistical analysis such as PCA, as a multivariate technique for analyzing quantitative data, and (iv) assess the potential carcinogenic health risk based on (Cd, Cu, Fe, Mn. Pb and Zn) metals concentrations based on dermal exposure pathway. All metals except Al, Ti, and Fe are typically treated as PHEs. Al, Ti, and Fe are generally reference/lithogenic metals, used to normalize anthropogenic enrichment (e.g., EF calculations). This is the first baseline study to analyze and estimate the current distribution of these elements’ loads in 11 sectors from coastal and non-coastal sediments along Egypt’s western Mediterranean coast, at depths ranging from 43 to 481 m.

## Materials and methods

### Study area and sampling

**Fig. 1:**
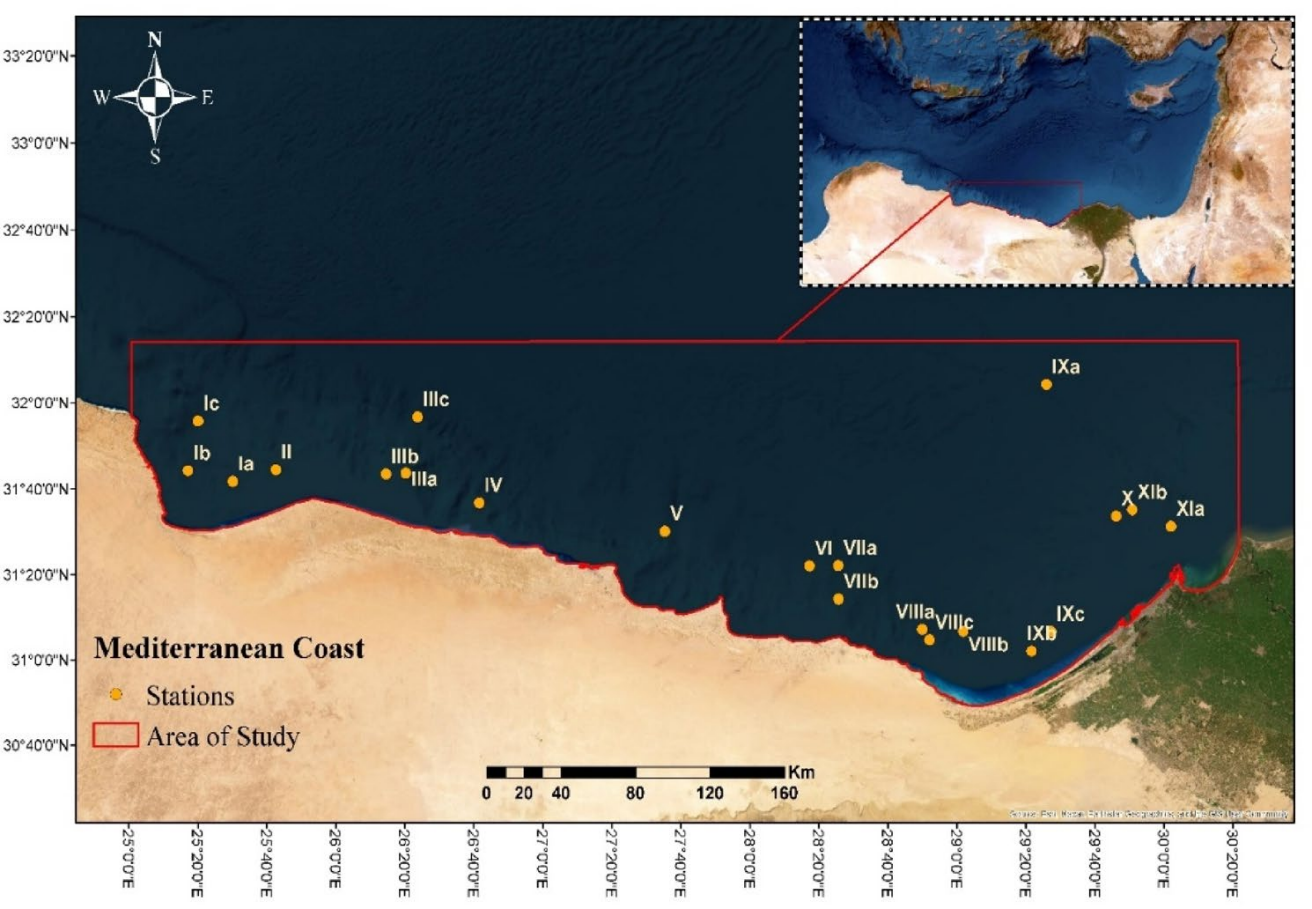
The map of sampling locations on the Mediterranean coast, Egypt (Software QGIS 3.18; https://www.filehorse.com/download-qgis/61739/).

Using a Van Veen grab sampler, surface samples were taken from 11 sectors along the western Mediterranean coast between El-Mex and Salloum in 2022. The depth positions ranged from 43 to 481 m, except in areas that were rocky or inaccessible (Fig. 1, Table S1). Each sample weighed around 100 g and was kept in sterile, wide-mouth glass vials covered with plastic bags. At each of the sample locations, three replicates were taken. The samples were safely delivered to the NIOF Laboratory in an icebox and kept at 4 °C until further examination. A list of sampling locations, including sampling depth and geographic coordinates, is provided in Table S1.

### Analytical methods of PHEs

After filtering the digested solutions with single-use 0.2 μm PTFE syringe filters (DISMIC-25HP, Advantech, Tokyo, Japan), the elements were determined using an ICP-MS analysis. In short, a 1:4:1 ratio of 1 ml H_2_O_2_, 4 ml HNO_3_, and 1 ml HCl was used to digest 0.2 g of the sediment samples. Samples were broken down for 90 min at 195 °C in a microwave digester. Following digestion, the materials were filtered and diluted with Milli-Q water to a volume of 10 mL^49^. Using the methods outlined in USEPA^49^, ICP-MS (iCAP, Thermo, Germany) evaluated the metal concentrations in these filtrates.

### Quality control and quality assurance (QC/QA)

The Quality Control and Quality Assurance (QC/QA) program was developed and implemented to ensure the production of reliable findings. As part of the QC/QA procedure, all measurements were performed in triplicate, and the results were reported as the mean value ± RSD. The data was deemed acceptable when the percentage difference between the three replicate samples and the RSD was less than 10%. Analytical results below the detection limit (BDL) were handled according to the contamination assessment guidelines provided by the EPA^49^. The certified reference materials, SRM 2702 (NIST, USA), were analyzed using the same method applied to the samples to ensure analytical reliability. The recoveries of all elements fell within the range between 97.1% and 100.9% (Table S2). The experiment was carried out three times, and the standard deviation values were ≤± 3.5.

### Contamination assessment

Two commonly used metrics to evaluate the level of anthropogenic PHEs pollution in environmental matrices such as soil and sediment are the Enrichment Factor (EF) and the Geoaccumulation Index (*I*_geo_). For the examined metal, *I*_geo_ was derived to describe the levels of PHEs pollution as shown in Eq. (1):

1 $$\:{\mathrm{I}}_{\mathrm{g}\mathrm{e}\mathrm{o}\:}=\:{\mathrm{l}\mathrm{o}\mathrm{g}}_{2}\left(\frac{{C}_{\mathrm{n}}}{1.5{B}_{\mathrm{n}}}\right)\:$$

where *C*_n_ represents the PHEs content detected (µg/g; dw), and *B*_n_ represents the background shale value (µg/g; dw)^51^. *I*_geo_ is divided into seven classes: class 0 (*I*_geo_ ≤ 0) refers to practically unpolluted sediment, class 1 (0 < *I*_geo_ < 1) refers to unpolluted to moderately polluted, class 2 (1 < *I*_geo_ < 2) moderately polluted, class 3 (2 < *I*_geo_ < 3) moderately to heavily polluted, class 4 (3 < *I*_geo_ < 4), heavily polluted, class 5 (4 < *I*_geo_ < 5) heavily to highly polluted and class 6 (*I*_geo_ > 5) extremely polluted^50^.

To distinguish between naturally occurring element concentrations and anthropogenic inputs, the EF compares a metal’s concentration to that of a reference element (such as Al, Ti, Fe, or Sc, which are stable and have low mobility). Equation (2) was used to interpret the *Enrichment factor (EF)* values for the investigated elements relative to the shale average, following Buat-Menard and Chesselet^52^.

2 $$\:\mathrm{E}\mathrm{F}=\frac{{\left(\frac{\mathrm{X}}{\mathrm{A}\mathrm{l}}\right)}_{\mathrm{s}\mathrm{e}\mathrm{d}\mathrm{i}\mathrm{m}\mathrm{e}\mathrm{n}\mathrm{t}}}{{\left(\frac{\mathrm{X}}{\mathrm{A}\mathrm{l}}\right)}_{\mathrm{s}\mathrm{h}\mathrm{a}\mathrm{l}\mathrm{e}}}$$

Where x/Al is the ratio of each HMs to Al.

Pollution load index (PLI).

*PLI* is a condensed approach for displaying the degree of sediment degradation in response to metal deposition. PLI can be calculated by Eq. (3).

3 $$\:PLI\:={(\:{\mathrm{C}\mathrm{F}}_{1}^{\mathrm{i}}.{\mathrm{C}\mathrm{F}}_{2}^{\mathrm{i}}.{\mathrm{C}\mathrm{F}}_{3}^{\mathrm{i}}\dots\:\dots\:{\mathrm{C}\mathrm{F}}_{\mathrm{n}}^{\mathrm{i}})}^{1/\mathrm{n}}\:$$

Where *CF* stands for contamination level and *n* for the number of elements present (µg/g; dw). *PLI* > 1 indicates deteriorated sediments, baseline values are *PLI* equal to 1, and ideal circumstances are *PLI* < 1^53^.

The overall effects of all elements are expressed by the contamination index (C_d_), which also evaluates the relative contamination of each element separately. This is the computation made using Eq. (4)^54^.

4 $$\:{\mathrm{C}}_{\mathrm{d}}=\sum\:_{\mathrm{i}=1}^{\mathrm{n}}{\mathrm{C}}_{\mathrm{f}\mathrm{i}}$$

Where *C*_fi_ can be calculated from Eq. (5):

5 $$\:{\mathrm{C}}_{\mathrm{f}\mathrm{i}}=\left(\frac{{\mathrm{C}}_{\mathrm{A}\mathrm{i}}}{{\mathrm{C}}_{\mathrm{N}\mathrm{i}}}\right)-1$$

The 1st value measured for the element in the sample is *C*_Ai_ (µg/g; dw), while the background value i^th^ for the element is *C*_Ni_ (used to calculate the element index and determine the sediment resources pollution degree to HMs). *C*_fi_ is the element’s contamination factor (*N* is the normative value). Three classifications are created from the resulting *C*_d_ values: high (*C*_d_ > 3), medium (*C*_d_ = 1–3), and low (*C*_d_ < 1).

Human health risk assessment.

Prolonged exposure to contaminated water and high levels of PHEs poses a severe health risk^55^. PHEs can be introduced into the body through ingestion, inhalation, or dermal absorption. In the current study, different indices were used to evaluate the risk associated with HMs for men, women, and children^56–58^. Equations (6–9) were used to compute the dermal risk for various populations^57,58^:

6 $$\:{CDI}_{dermal}\:=\:\frac{C\:\times\:\:SA\:\times\:SL\:\times\:\:ABF\:\times\:\:ED\:\times\:\:EF\:}{BW\:\times\:\:AT}$$

where *CDI*_Dermal_ is the chronic daily intake, *C* is the concentration of PHEs in the sediment (µg/g; dw), *SA* is the exposed area of the skin, *SL* is the skin adhesion factor, *ABF* is the skin adsorption factor, *EF* is the exposure frequency, *ED* is the exposure duration, *BW* is the average body weight (kg), and *AT* is the average time (day). ATSDR^59^and Shetty et al.^60^offer suggested default values.

7 $$\:{HQ}_{\mathrm{d}\mathrm{e}\mathrm{r}\mathrm{m}\mathrm{a}\mathrm{l}\:}=\frac{{\mathrm{C}\mathrm{D}\mathrm{I}}_{\mathrm{d}\mathrm{e}\mathrm{r}\mathrm{m}\mathrm{a}\mathrm{l}}}{{\mathrm{R}\mathrm{f}\mathrm{D}}_{\mathrm{d}\mathrm{e}\mathrm{r}\mathrm{m}\mathrm{a}\mathrm{l}}}$$

*Where HQ hazard quotient*,* RFD reference dose*.

8 $$\:HI\:=\:\sum\:_{\mathrm{i}=1}^{\mathrm{n}}\:{HQ}_{\mathrm{i}}$$

Where the HI hazard index.

9 $$\:{CR}_{\mathrm{d}\mathrm{e}\mathrm{r}\mathrm{m}\mathrm{a}\mathrm{l}\:}=\:{\mathrm{C}\mathrm{D}\mathrm{I}}_{\mathrm{d}\mathrm{e}\mathrm{r}\mathrm{m}\mathrm{a}\mathrm{l}\:}\times\:\mathrm{C}\mathrm{S}\mathrm{F}$$

*Where CR*_Dermal_ refers to carcinogenic risk through dermal absorption of elements in sediment, and CSF is the cancer slope factor^61^.

### Statistical analysis and identification of sources of HMs

SPSS Version 19 was used for the multivariate analysis and the correlation analysis in the statistical analysis of the present study.

## Results and discussion

### Distribution of different elements and contamination indices of toxic metals

**Fig. 2:**
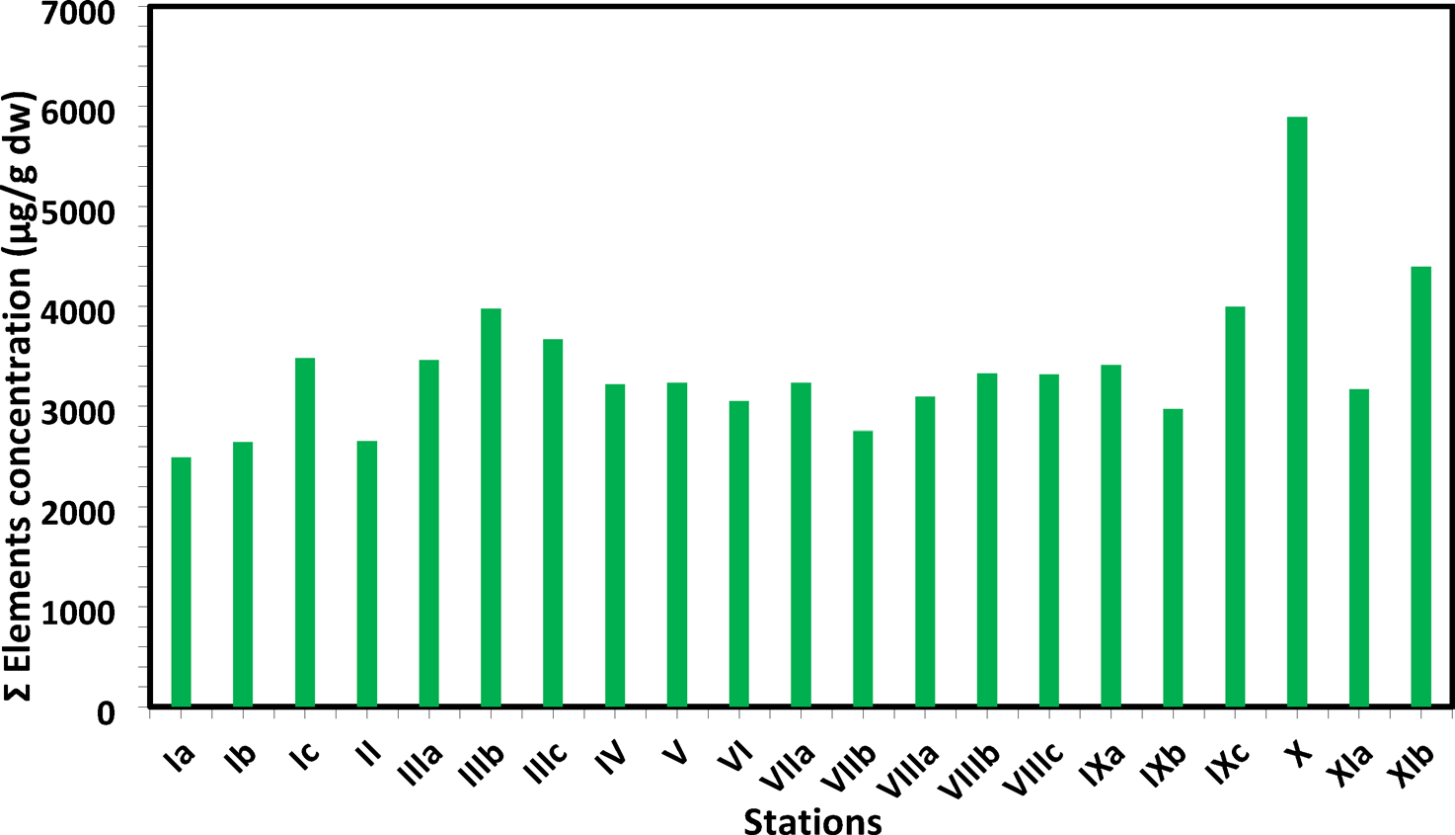
The average concentrations of total elements (µg/g, d.w.) in collected sediment samples.

**Fig. 3:**
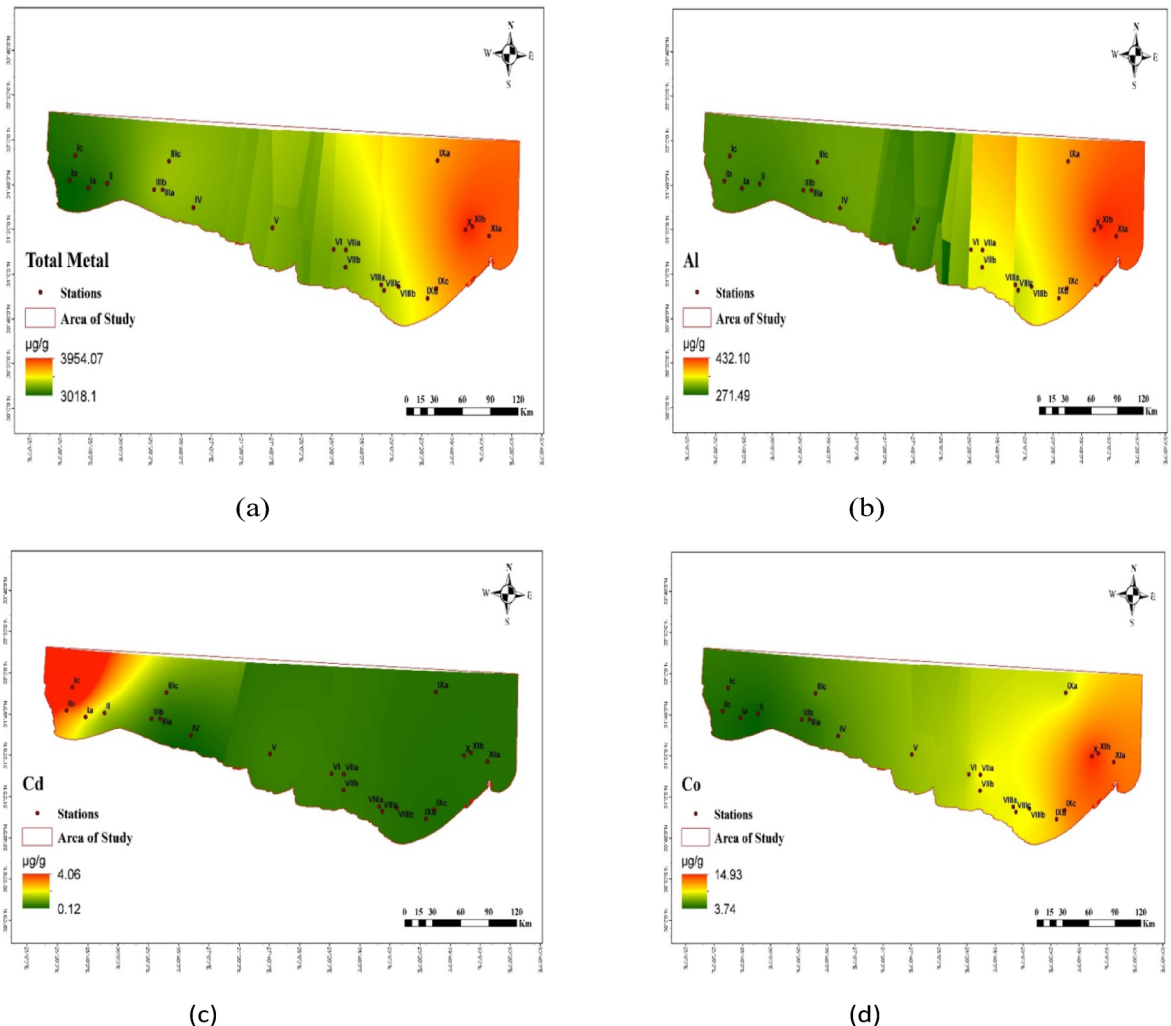

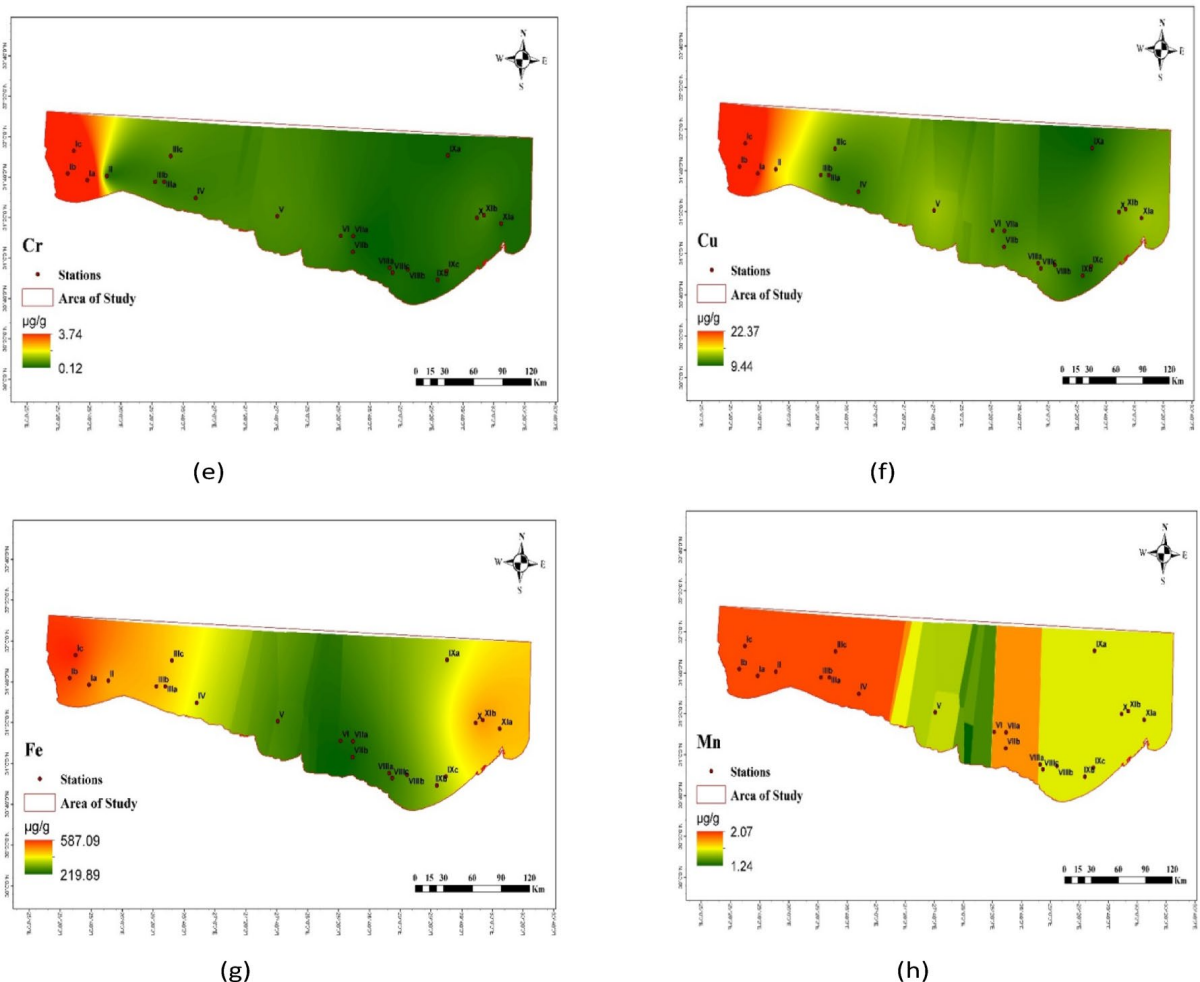

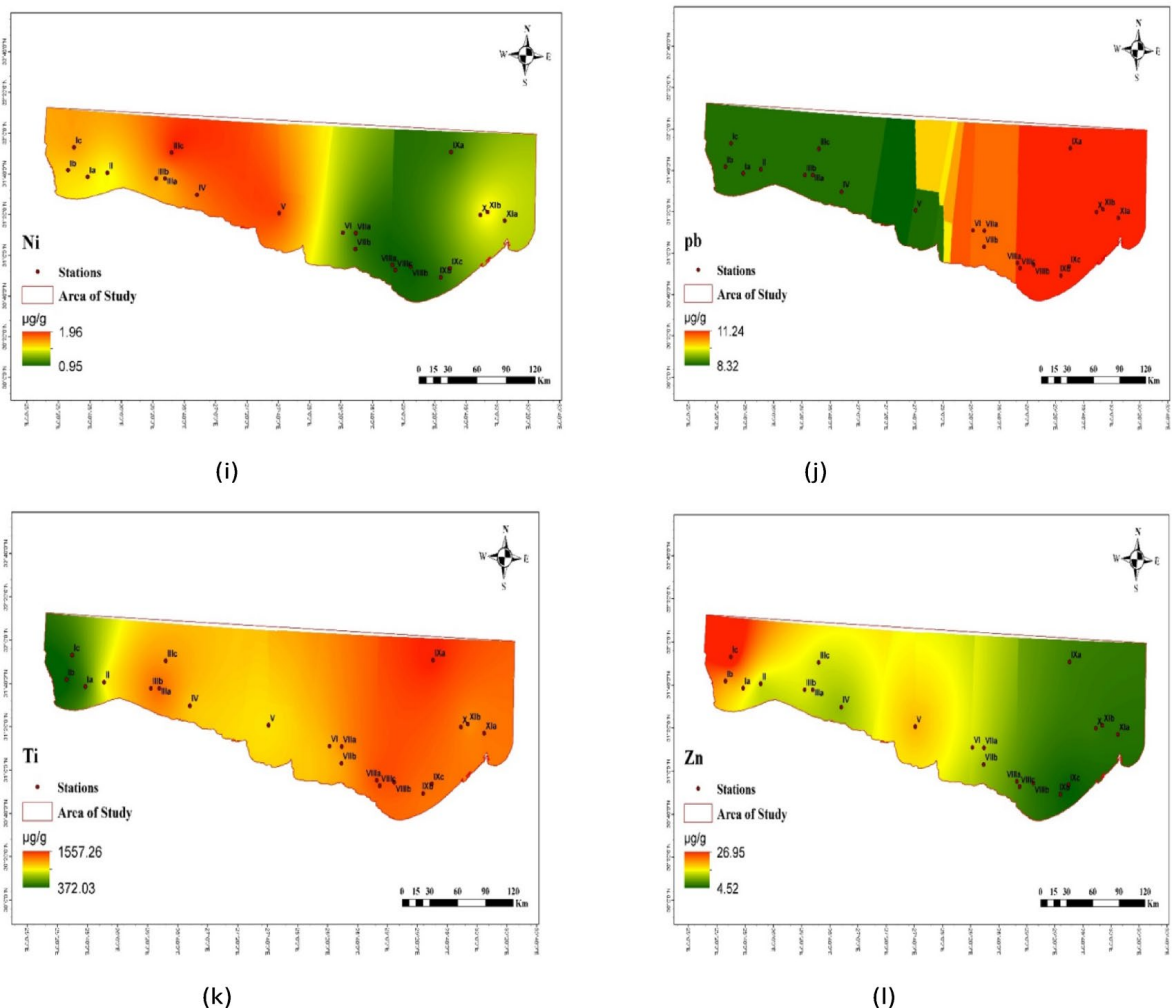
The distribution of elements (µg/g, d.w.) in the collected sediment samples.

The distribution of PHE concentrations (µg/g) in the investigated sediments is displayed in Table 1; Figs. 2 and 3. PHEs ranged from 2492.95 µg/g at Salloum (Ia) Station to 5890.61 µg/g dry weight (dw) at Sedi Krrir (X) Station, with an average of 3402.69 ± 737.29 µg/g (Fig. 2). Ti had the highest average value of 1710.09 µg/g, while In had the lowest average value of 0.01 µg/g (Table 1). The elements found at each site varied considerably. This may have human origins in the industrialized, heavily populated nations surrounding the research area. The discharge of oil terminals, shipping, agricultural drains, industrial waste, domestic sewage, organic matter, fertilizers, and pesticides has contaminated the Egyptian Mediterranean coast^62^. Due to nearby industrial operations and sewage drains that discharged into the coastal sea via El Mex pumps, Sedi Krrir Station has the highest concentration for all recorded elements. The remaining stations are located in the western parts of Egypt’s Mediterranean coast, away from densely populated areas.

**Table 1:** Concentrations of different elements (µg/g; dw) in sediment samples.

Element	Li	B	Na	Mg	Al	K	Ca	Ti	Cr	Mn	Fe	Co
Location												
Ia	0.15	2.08	653.53	687.34	227.63	53.39	143.90	143.97	3.77	1.54	513.89	3.78
Ib	0.25	2.54	657.72	525.52	324.91	47.20	110.47	310.51	2.40	1.85	607.65	3.86
Ic	0.41	1.53	813.76	589.87	382.16	51.15	197.71	365.32	2.50	3.79	965.82	4.91
II	0.12	1.72	514.15	297.16	110.61	34.13	137.21	1338.24	0.21	0.41	151.47	2.73
IIIa	0.13	1.58	414.34	463.87	77.35	29.28	178.36	1710.91	0.26	0.44	475.96	2.12
IIIb	0.31	1.74	631.84	699.57	284.15	54.76	137.28	1360.99	0.31	3.13	652.49	5.52
IIIc	0.41	1.31	503.93	578.74	551.77	60.84	114.21	1191.77	0.42	2.41	424.88	7.03
IV	0.31	1.65	858.00	447.64	396.95	56.62	93.52	982.59	0.32	0.99	327.16	5.58
V	0.36	2.46	745.59	593.36	475.72	79.22	102.15	989.63	0.40	3.84	183.63	7.23
VI	0.29	1.07	509.10	638.92	330.41	45.54	109.62	1099.17	0.29	3.58	267.03	4.61
VIIa	0.13	2.31	518.27	428.44	179.73	50.77	148.72	1426.53	0.14	0.48	166.62	14.33
VIIb	0.24	1.23	477.22	591.40	208.24	46.03	107.42	1045.83	0.17	1.68	136.38	8.53
VIIIa	0.14	1.06	555.81	215.93	189.73	53.13	172.29	1665.18	0.13	0.28	191.55	6.68
VIIIb	0.22	1.37	603.22	595.64	181.57	53.77	148.74	1475.79	0.16	0.46	217.61	6.73
VIIIc	0.27	1.30	708.64	490.99	291.84	53.56	134.52	1346.77	0.18	0.70	232.50	11.82
IXa	0.19	0.86	572.44	352.68	236.47	45.07	171.86	1657.68	0.14	0.36	202.73	5.17
Ixb	0.20	0.94	492.91	534.40	160.89	42.86	133.54	1312.47	0.14	0.84	211.58	8.57
Ixc	0.40	0.86	977.42	478.93	468.67	57.73	127.99	1396.34	0.24	1.17	430.37	15.76
X	0.45	0.79	719.43	466.20	699.88	62.68	128.88	1465.05	0.31	1.81	639.04	20.66
Xia	0.26	0.85	600.96	282.96	302.65	57.49	138.48	1411.51	0.18	0.65	308.27	11.25
Xib	0.76	0.77	666.45	383.42	1164.25	52.63	66.73	1018.78	0.37	7.34	977.12	14.90
Average	0.28	1.43	628.32	492.52	345.03	51.8	133.50	1176.91	0.62	1.80	394.46	8.18
SD	0.15	0.56	140.01	132.37	241.67	10.25	30.81	435.74	0.98	1.74	252.98	4.88
Min	0.12	0.77	414.34	215.93	77.35	29.28	66.73	143.97	0.13	0.28	136.38	2.12
Max	0.76	2.54	977.42	699.57	1164.25	79.22	197.71	1710.91	3.77	7.34	977.12	20.66

Element	Ni	Cu	Zn	Ga	Se	Sr	Ag	Cd	In	Ba	Pb	Bi
Location												
Ia	1.61	27.17	13.74	0.86	0.52	1.77	4.02	0.31	0.06	2.71	4.91	0.32
Ib	1.45	18.22	11.87	1.64	0.22	1.47	4.47	0.52	0.06	4.37	5.67	0.03
Ic	1.95	31.42	40.89	2.91	0.21	0.87	7.07	7.98	0.07	2.73	5.26	0.19
II	1.11	5.34	6.51	0.48	0.22	3.38	46.79	0.14	0.01	2.36	2.28	0.10
IIIa	1.04	2.19	12.79	0.52	0.23	4.37	81.27	0.10	0.01	3.30	0.54	0.03
IIIb	2.03	16.12	14.88	1.03	0.30	1.65	96.36	0.31	0.06	4.50	5.82	0.23
IIIc	2.41	11.30	10.98	2.46	0.29	1.16	191.53	0.23	0.02	4.78	7.13	0.14
IV	1.79	9.18	7.75	1.93	0.20	1.59	9.62	0.15	0.03	7.64	6.48	0.47
V	2.20	15.37	20.46	1.27	0.22	1.54	2.65	0.46	0.03	3.45	2.65	0.42
VI	1.27	11.16	12.44	1.45	0.21	1.11	4.57	0.41	0.03	3.00	4.82	0.10
VIIa	0.77	8.35	13.2	0.66	0.24	2.93	212.48	0.24	0.06	3.78	55.64	1.04
VIIb	1.04	10.20	8.71	0.67	0.22	3.15	104.94	0.33	0.07	0.5	0.57	0.03
VIIIa	0.85	11.53	6.92	0.66	0.23	4.42	13.09	0.30	0.03	0.73	3.87	0.10
VIIIb	0.85	10.46	4.85	1.15	0.32	2.62	14.67	0.21	0.04	1.29	4.87	0.06
VIIIc	1.11	14.23	7.60	1.36	0.55	2.73	12.01	0.32	0.04	0.92	5.27	0.04
Ixa	0.87	7.16	6.13	0.82	4.21	4.18	113.79	0.27	0.02	6.34	23.79	0.59
Ixb	0.85	6.84	4.42	0.86	0.23	2.11	50.03	0.20	0.01	5.77	3.62	0.02
Ixc	1.17	9.77	3.58	2.15	0.27	2.76	7.97	0.22	0.08	5.34	6.79	0.70
X	1.45	12.82	8.10	4.63	0.21	2.72	1638.11	0.26	0.46	0.90	13.18	2.59
Xia	1.17	14.74	5.98	1.42	0.20	3.53	19.58	0.29	0.06	0.99	7.65	0.41
Xib	2.00	12.22	4.20	4.77	0.19	1.44	9.95	0.20	0.05	0.76	4.87	0.07
Average	1.38	12.66	10.76	1.60	0.45	2.45	125.95	0.64	0.06	3.15	8.37	0.37
SD	0.50	6.72	8.12	1.22	0.87	1.10	351.99	1.69	0.09	2.08	11.88	0.58
Min	0.77	2.19	3.58	0.48	0.19	0.87	2.65	0.10	0.01	0.50	0.54	0.02
Max	2.41	31.42	40.89	4.77	4.21	4.42	1638.11	7.98	0.46	7.64	55.64	2.59

Particularly in Sedi Krrir and El Mex areas, which exhibit the highest total PHEs, agricultural effluents and untreated wastewater released from various plants of the paper, food processing, textile, and fertilizer industries are responsible for the highest values of Cu, Ni, and Co (Fig. 2). Salloum and Marsa Matrouh stations had the lowest average total PHEs concentrations of any station, which may be due to less anthropogenic activity in these areas than in other places^62^.

### Ecological implications

**Fig. 4:**
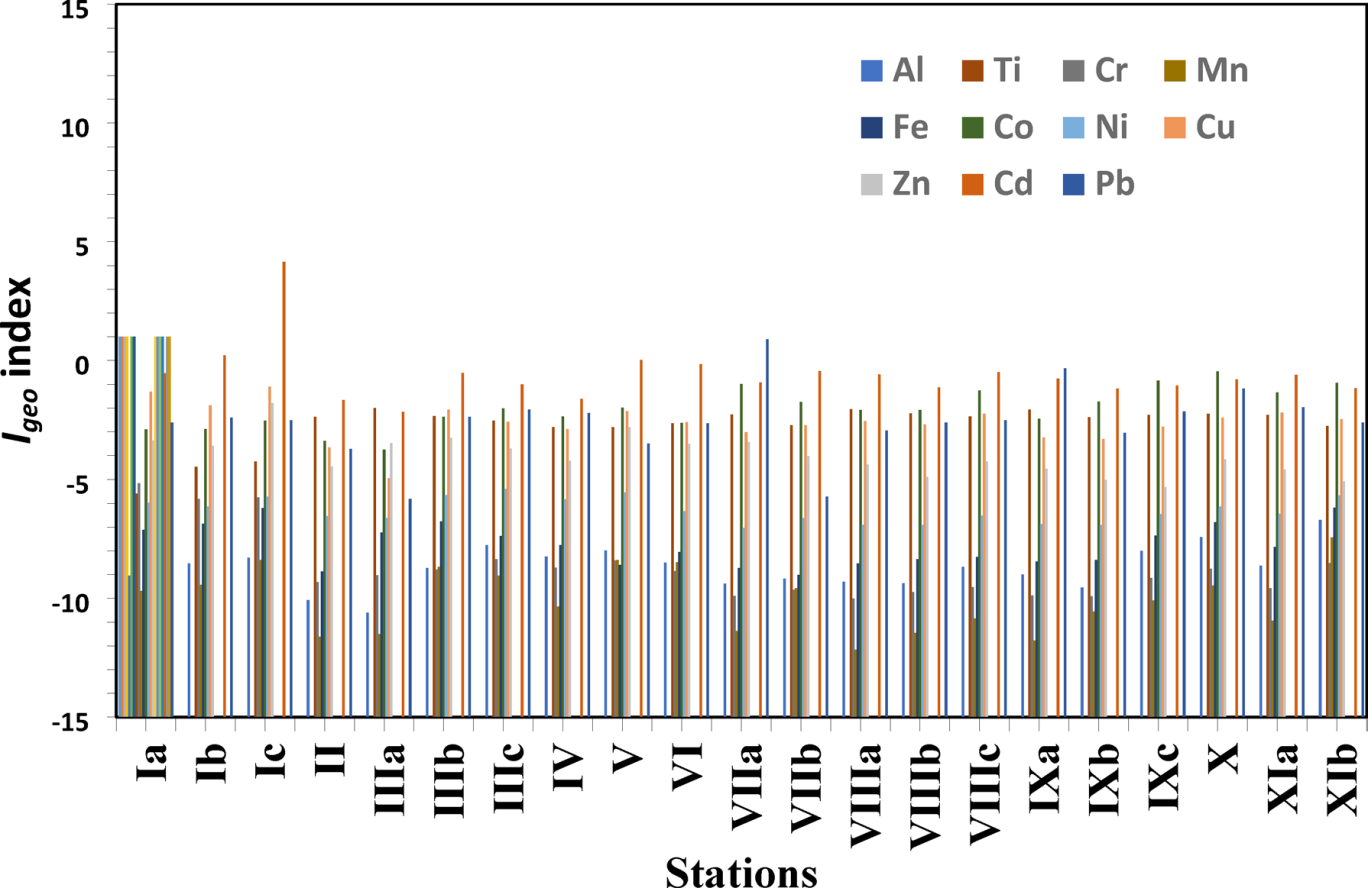
The *I*_geo_ for the investigated sediment samples.

Ecological implications refer to the positive or negative effects that events or actions (such as pollution, invasive species, or climate change) have on living organisms and their environment. These effects include disrupting food webs, natural balances, and essential ecosystem services such as pollination and clean water, which often result in species shifts, biodiversity loss, and decreased ecosystem resilience. These effects have a profound impact on every aspect of the web of life, from individual species to vast landscapes, and they also affect human welfare. The *I*_geo_ in the studied sediments are shown in Table 2; Fig. 4. The level of metal enrichment was given according to the Igeo classification^63^. They stated that *I*_geo_ ≤ 0; 0 ≤ *I*_geo_ ≤ 1; 1 ≤ *I*_geo_ ≤ 2; 2 ≤ *I*_geo_ ≤ 3; 3 ≤ *I*_geo_ ≤ 4; 4 ≤ *I*_geo_ ≤ 5; and *I*_geo_ > 6 are classified as class (0): unpolluted; class (1): unpolluted to moderately polluted; class (2): moderately polluted; class (3): moderately to strongly polluted; class (4): strongly polluted; class (5): strongly to extremely polluted; and class (6) extremely polluted. The *I*_geo_ data indicated that all the studied sediments were unpolluted, with an *I*_geo_ class 0 for the following elements: Al, Ti, Cr, Mn, Fe, Co, Ni, Cu, Zn. For locations Ib and Ic, Cd was recorded as class 1 and class 4, respectively. Station VIIa is classified as class 1 for Pb (Table 2).

**Fig. 5:**
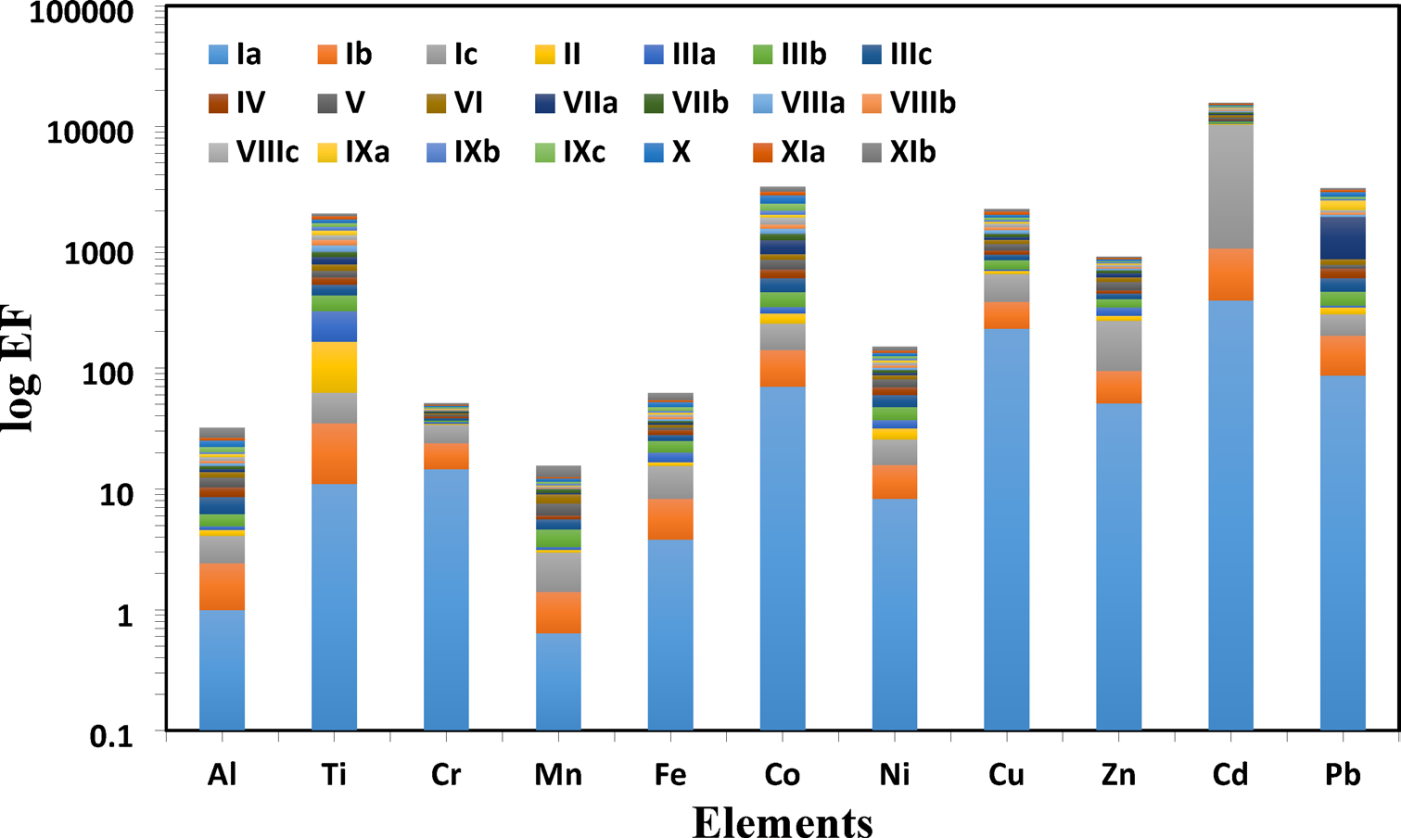
The enrichment factor (EF) of the collected sediment samples.

**Fig. 6:**
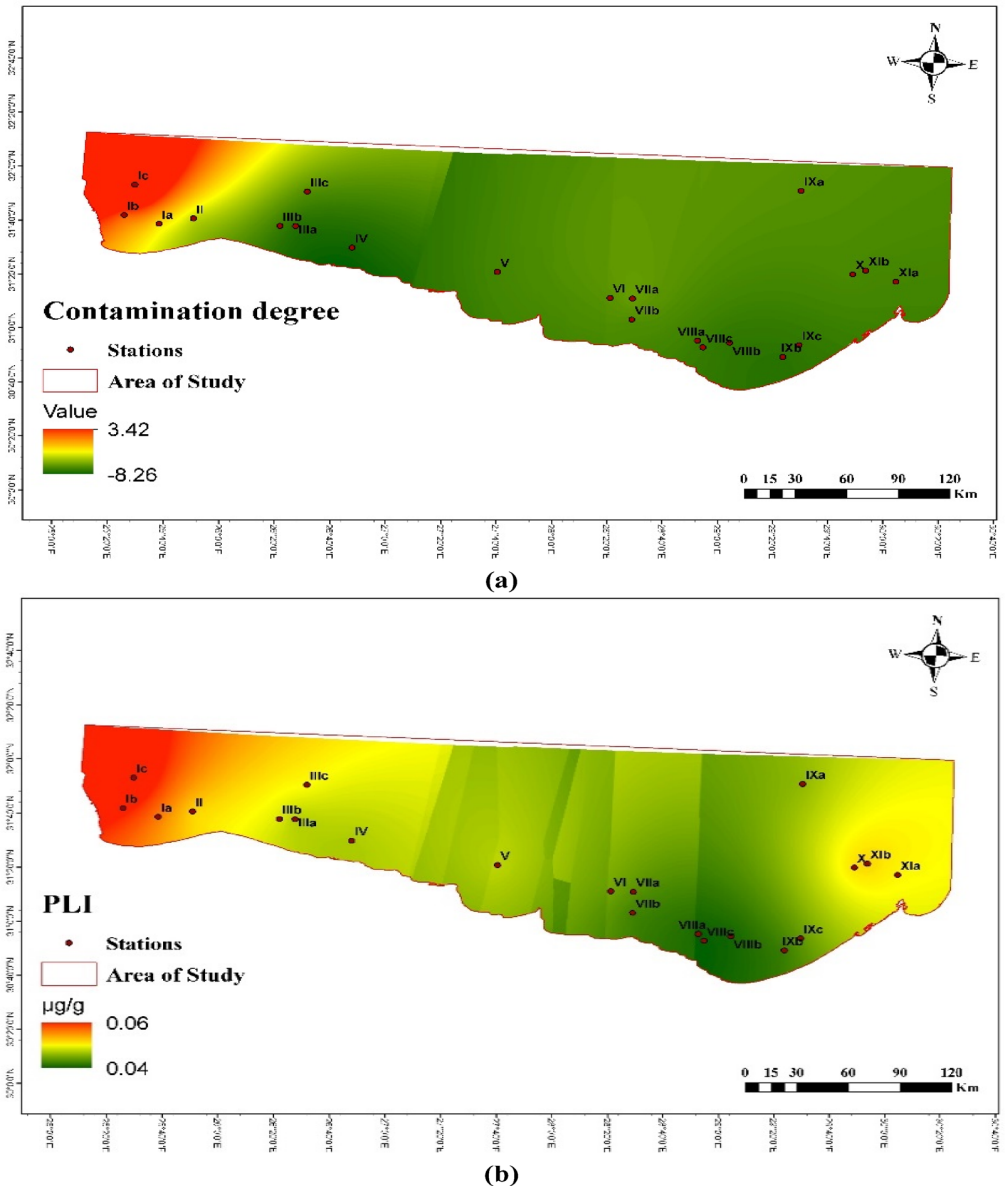
The contamination degree (**a**) and the pollution loading index (**b**) of the studied metals.

The enrichment factor (*EF*) in the studied sediments is shown in Table 3; Fig. 5. Aluminum was used as a normalizing element in the calculation of the enrichment factor (EF), as stated by Birth^64^, to assess the anthropogenic contribution (Table 3). Cd shows the highest *EF* value among all the studied stations, with the highest value at Salloum station (Ic). Mn shows the lowest *EF* value with the lowest value at station VIIIa. Figure 6 shows the *C*_d_ and the *PLI* of the studied metals. The calculated *PLI* and *C*_d_ in the studied sediments are shown in Table 4. The values of *PLI* extended from 0.022 at Sedi Branny station to 0.104 at Salloum station (Table 4), indicating no pollution in any of the coastal deposits. The *C*_d_ revealed the highest degree of contamination, with a score of 18.40, at the Salloum station.

Extremely high enrichment factors (EF > 5000) are generally categorized as “extremely high enrichment” or “extremely severe enrichment” and typically indicate a significant, concentrated source of anthropogenic (human-caused) pollution. This consequently means there is a need for future explanation to clarify the reason for this substantial increase. They should be carefully verified.

**Table 2:** *I*_geo_ for the investigated sediment samples.

Location	Ia	Ib	Ic	II	IIIa	IIIb	IIIc	IV	V	VI
Element										
Al	−9.04	−8.53	−8.29	−10.08	−10.6	−8.72	−7.76	−8.24	−7.98	−8.5
Ti	−5.58	−4.47	−4.24	−2.37	−2.01	−2.34	−2.53	−2.81	−2.8	−2.65
Cr	−5.16	−5.81	−5.75	−9.32	−9.03	−8.79	−8.35	−8.71	−8.4	−8.85
Mn	−9.69	−9.43	−8.39	−11.61	−11.5	−8.67	−9.05	−10.34	−8.38	−8.48
Fe	−7.11	−6.86	−6.20	−8.87	−7.22	−6.76	−7.38	−7.76	−8.59	−8.05
Co	−2.91	−2.88	−2.54	−3.39	−3.75	−2.37	−2.02	−2.35	−1.98	−2.63
Ni	−5.98	−6.14	−5.71	−6.53	−6.61	−5.65	−5.4	−5.83	−5.54	−6.33
Cu	−1.31	−1.89	−1.10	−3.66	−4.94	−2.07	−2.58	−2.88	−2.13	−2.6
Zn	−3.37	−3.59	−1.80	−4.45	−3.48	−3.26	−3.7	−4.2	−2.8	−3.52
Cd	−0.54	0.22	4.15	−1.66	−2.16	−0.53	−1.00	−1.62	0.02	−0.15
Pb	−2.61	−2.40	−2.51	−3.72	−5.81	−2.37	−2.07	−2.21	−3.50	−2.64

Location	VIIa	VIIb	VIIIa	VIIIb	VIIIc	Ixa	Ixb	Ixc	X	Xia	Xib
Element											
Al	−9.38	−9.17	−9.3	−9.37	−8.68	−8.99	−9.54	−8	−7.42	−8.63	−6.69
Ti	−2.27	−2.72	−2.05	−2.23	−2.36	−2.06	−2.39	−2.3	−2.24	−2.29	−2.76
Cr	−9.89	−9.64	−10.01	−9.73	−9.53	−9.88	−9.91	−9.14	−8.75	−9.57	−8.52
Mn	−11.37	−9.57	−12.17	−11.45	−10.84	−11.77	−10.56	−10.09	−9.46	−10.94	−7.44
Fe	−8.73	−9.02	−8.53	−8.35	−8.25	−8.45	−8.39	−7.36	−6.79	−7.84	−6.18
Co	−0.99	−1.74	−2.09	−2.08	−1.27	−2.46	−1.73	−0.85	−0.46	−1.34	−0.94
Ni	−7.04	−6.62	−6.91	−6.9	−6.52	−6.88	−6.91	−6.45	−6.13	−6.44	−5.67
Cu	−3.02	−2.73	−2.55	−2.69	−2.25	−3.24	−3.3	−2.79	−2.4	−2.19	−2.47
Zn	−3.43	−4.03	−4.37	−4.88	−4.23	−4.54	−5.01	−5.31	−4.14	−4.57	−5.08
Cd	−0.92	−0.44	−0.59	−1.13	−0.49	−0.76	−1.18	−1.06	−0.79	−0.61	−1.17
Pb	0.89	−5.72	−2.95	−2.62	−2.51	−0.33	−3.05	−2.14	−1.19	−1.97	−2.62

**Table 3:** The enrichment factor (EF) for the collected sediment samples.

Location	Ia	Ib	Ic	II	IIIa	IIIb	IIIc	IV	V	VI
Element										
Al	1	1.43	1.68	0.49	0.34	1.25	2.42	1.74	2.09	1.45
Ti	11	23.72	27.91	102.24	130.71	103.98	91.05	75.07	75.61	83.98
Cr	14.71	9.38	9.76	0.82	1.01	1.19	1.62	1.26	1.56	1.14
Mn	0.64	0.77	1.57	0.17	0.18	1.29	1.00	0.41	1.59	1.48
Fe	3.83	4.52	7.19	1.13	3.54	4.86	3.16	2.44	1.37	1.99
Co	69.9	71.45	90.84	50.46	39.21	102.18	130	103.14	133.75	85.25
Ni	8.34	7.48	10.06	5.72	5.38	10.47	12.45	9.25	11.36	6.55
Cu	212.18	142.32	245.36	41.7	17.13	125.91	88.23	71.66	120.03	87.12
Zn	50.83	43.92	151.28	24.07	47.32	55.04	40.62	28.68	75.67	46.02
Cd	363.16	612.68	9351.85	166.35	118.32	365.5	263.58	171.03	534.19	474.45
Pb	86.35	99.6	92.38	40.05	9.4	102.29	125.29	113.85	46.51	84.66

Location	VIIa	VIIb	VIIIa	VIIIb	VIIIc	IXa	IXb	IXc	X	Xia	XIb
Element											
Al	0.79	0.91	0.83	0.8	1.28	1.04	0.71	2.06	3.07	1.33	5.11
Ti	108.99	79.9	127.22	112.75	102.89	126.65	100.27	106.68	111.93	107.84	77.84
Cr	0.55	0.66	0.51	0.62	0.71	0.56	0.55	0.93	1.22	0.69	1.43
Mn	0.2	0.7	0.11	0.19	0.29	0.15	0.35	0.48	0.75	0.27	3.04
Fe	1.24	1.02	1.43	1.62	1.73	1.51	1.58	3.2	4.76	2.3	7.28
Co	265.02	157.78	123.61	124.47	218.56	95.67	158.59	291.46	382.18	208.03	275.68
Ni	4	5.36	4.39	4.41	5.74	4.49	4.37	6.03	7.5	6.05	10.34
Cu	65.21	79.64	90.02	81.67	111.16	55.93	53.44	76.28	100.09	115.13	95.43
Zn	48.82	32.23	25.58	17.93	28.11	22.66	16.35	13.26	29.96	22.14	15.54
Cd	278.81	387.76	350.27	240.15	376.04	311.61	231.95	253.04	305.75	344.41	234.29
Pb	977.78	10.02	68	85.54	92.62	418.11	63.61	119.28	231.51	134.46	85.49

**Table 4:** The *PLI* and contamination index (*C*_d_) in the collected marine sediment samples.

Parameter	PLI	C_d_
Location		
Ia	0.052	−7.70
Ib	0.057	−8.11
Ic	0.104	18.40
II	0.024	−8.77
IIIa	0.022	−8.94
IIIb	0.058	−7.52
IIIc	0.057	−7.84
IV	0.041	−8.36
V	0.056	−7.15
VI	0.049	−7.52
VIIa	0.044	−5.02
VIIb	0.031	−7.85
VIIIa	0.031	−7.74
VIIIb	0.032	−8.09
VIIIc	0.042	−7.33
IXa	0.036	−7.05
IXb	0.030	−8.20
IXc	0.045	−7.52
X	0.065	−6.65
XIa	0.043	−7.31
XIb	0.066	−7.70

### Human health risk assessment

The human risk assessment for adults and children (carcinogenic and non-carcinogenic) through the dermal adsorption pathway for marine sediments was calculated using the *CDI*,* HQ*,* CR*,* and HI* calculations. Tables (S3–S5) present the CDI (µg/g/Day) values for adults, adolescents, and children. Based on *CDI* readings, the sequence of PHEs was Fe > Mn > Pb > Cu > Zn. Table 5 displays the *HI* and *CR* values for adults, adolescents, and children. According to the values of *HQ* across the various demographics, children have values 3–4 times higher than those of the adult population (Table S6). PHEs were recorded among human groups in the following order: Cd > Cu > Fe > Mn > Pb > Zn. The carcinogenic risk (CR) of Cd and Pb via the dermal route was calculated using their carcinogenic slope factor. The *CR* value ranges for Cd were 4.43 × 10^–4^ – 1.76 × 10^–2^ for males, 2.01 × 10^–4^ – 1.59 × 10^–2^ for females, and 7.42 × 10^–4^ – 5.87 × 10^–2^ for children, suggesting a risk of cancer > 10^–6^^65^. The *CR* ranges for Pb in children (4.21 × 10^–7^ – 3.32 × 10^–5^), males (6.78 × 10^–7^ – 7.05 × 10^–5^), and females (2.23 × 10^–6^ – 2.32 × 10^–4^), suggesting risk of cancer < 10^–6^.

**Table 5:** *HI* and *CR* values for males, females, and children in the collected sediment samples.

Parameter	Hazard Index (HI)			Carcinogenic risk (CR)					
	Adult		Children	Adult				Children	
	Males	Females		Males		Females			
				Cd	Pb	Cd	Pb	Cd	Pb
Locations									
Ia	4.09E−01	4.09E−01	1.51E + 00	6.94E−04	6.23E−06	6.16E−04	2.05E−05	2.28E−03	1.29E−06
Ib	4.51E−01	4.51E−01	1.67E + 00	1.17E−03	7.19E−06	1.04E−03	2.36E−05	3.84E−03	2.18E−06
Ic	1.54E + 00	1.54E + 00	5.70E + 00	1.79E−02	6.66E−06	1.59E−02	2.19E−05	5.87E−02	3.32E−05
II	1.51E−01	1.51E−01	5.57E−01	3.18E−04	2.89E−06	2.82E−04	9.49E−06	1.04E−03	5.91E−07
IIIa	1.26E−01	1.26E−01	4.65E−01	2.26E−04	6.78E−07	2.01E−04	2.23E−06	7.42E−04	4.21E−07
IIIb	4.31E−01	4.31E−01	1.60E + 00	6.98E−04	7.38E−06	6.20E−04	2.42E−05	2.29E−03	1.30E−06
IIIc	4.14E−01	4.14E−01	1.53E + 00	5.03E−04	9.04E−06	4.47E−04	2.97E−05	1.65E−03	9.37E−07
IV	3.52E−01	3.52E−01	1.30E + 00	3.27E−04	8.21E−06	2.90E−04	2.70E−05	1.07E−03	6.08E−07
V	2.54E−01	2.54E−01	9.38E−01	1.02E−03	3.36E−06	9.07E−04	1.10E−05	3.35E−03	1.90E−06
VI	3.25E−01	3.25E−01	1.20E + 00	9.06E−04	6.11E−06	8.05E−04	2.01E−05	2.98E−03	1.69E−06
VIIa	2.19E + 00	2.19E + 00	8.09E + 00	5.32E−04	7.05E−05	4.73E−04	2.32E−04	1.75E−03	9.91E−07
VIIb	1.27E−01	1.27E−01	4.70E−01	7.40E−04	7.23E−07	6.58E−04	2.37E−06	2.43E−03	1.38E−06
VIIIa	2.59E−01	2.59E−01	9.56E−01	6.69E−04	4.91E−06	5.95E−04	1.61E−05	2.20E−03	1.25E−06
VIIIb	2.84E−01	2.84E−01	1.05E + 00	4.59E−04	6.17E−06	4.08E−04	2.03E−05	1.51E−03	8.54E−07
VIIIc	3.31E−01	3.31E−01	1.22E + 00	7.18E−04	6.68E−06	6.38E−04	2.20E−05	2.36E−03	1.34E−06
IXa	9.92E−01	9.92E−01	3.66E + 00	5.95E−04	3.02E−05	5.29E−04	9.91E−05	1.96E−03	1.11E−06
IXb	2.23E−01	2.23E−01	8.25E−01	4.43E−04	4.59E−06	3.94E−04	1.51E−05	1.46E−03	8.25E−07
IXc	3.91E−01	3.91E−01	1.44E + 00	4.83E−04	8.61E−06	4.30E−04	2.83E−05	1.59E−03	9.00E−07
X	6.84E−01	6.84E−01	2.53E + 00	5.84E−04	1.67E−05	5.19E−04	5.49E−05	1.92E−03	1.09E−06
XIa	4.30E−01	4.30E−01	1.60E + 00	6.58E−04	9.70E−06	5.85E−04	3.19E−05	2.16E−03	1.22E−06
XIb	4.21E−01	4.21E−01	1.56E + 00	4.47E−04	6.17E−06	3.98E−04	2.03E−05	1.47E−03	8.33E−07

### Ecotoxicological effects of PHEs

It is evident from comparing our results in Table 1 with those in Table S7 that the recorded concentrations of Ni, Cd, and Zn do not exceed the TEL, PEL, ERL, and ERM values for all sampling stations. While stations Ia, Ib, and Ic exceeded the TEL level for Cu, station Ic also exceeded both the PEL and TEL for Cu. The SQGs created for marine and estuarine ecosystems were used to establish the Ecotoxicological effects of PHEs pollution in sediment^66^. When ERL and TEL are present in quantities below these levels, detrimental impacts on the fauna living in the sediment are improbable. Chemical concentrations above those where harmful effects are expected to occur were represented by ERM and PEL^66^. The threshold effect concentration (TEC, or the concentration at which toxicity may be detected) and the probable effect concentration (PEC, or the concentration above which toxicity is commonly observed) were both described by MacDonald et al.^67^. Sediment contamination is assessed using a variety of indicators and compared to effect-based SQGs. Numerical SQGs have been used to detect pollutants of concern in aquatic ecosystems (Table S7). Sediments were classified as non-contaminated, moderately polluted, or highly polluted based on the USEPA’s SQGs^68–77^. The studied sediment is not contaminated with (Cu, Zn, and Ni) according to SQG. Generally, the sensitivity analyses consistently demonstrate that the metal concentrations in the exposure medium and the exposure rates (e.g., ingestion rate) are the most critical factors in human health risk evaluations for PHEs^78^.

### Correlation matrix and source identification of elements

The correlation structure between various elements was assessed using Pearson’s correlation matrix. Mn has a strong relationship with Fe (0.673), Ni (0.702) and Ga (0.645) (Table S8). Al has strong relationships with Mn (0.789), Fe (0.628), Co (0.564) and Ni (0.598). Cu and Zn exhibit comparable geochemical behavior and co-occur in minerals that form rocks^79,80^; this was confirmed by a strong correlation between them (0.692). One of the global environmental problems is metal pollution. Kachoueiyan et al.^81^ aimed to assess the concentration, potential ecological danger, and source of Al, As, Co, Cr, Cu, Fe, Mn, Ni, and Zn in the sediments and water of the Gomishan wetland. As was the only contaminant found in sediments, according to sediment contamination indexes. There were no significant ecological risks associated with the metals under investigation, as indicated by the possible environmental risk index (RI), toxic risk index (TRI), and chemical speciation assessments. PCA and correlation analysis revealed that all metals examined in the Gomishan wetland sediments originated from natural sources. The water quality was unsuitable for aquatic life due to the presence of harmful components, as indicated by the HPI and HEI indices. PCA is considered one of the most significant methods for component analysis (Table S9 and Fig. S1). It is also a useful tool for modelling, pattern recognition, classification, and other data analysis tasks^82,83^. When the two axes of the ordination function are shown, data from an experimental system with comparable properties are plotted close together, whereas data from a system with different properties are plotted far apart^84^. The PC1 group accounted for 30.61% of the total variance, with a high loading of Li, Mn, Ga, Fe, and Al, indicating that these elements were correlated at all stages (Table S9). The PC2 component accounted for 21.565% of the total variance with a high loading of Co (− 0.829). The PC3 component accounted for 11.72%, correlated with Ca (0.710).

In certain instances, the study’s results were considerably lower than those in other Mediterranean nations (Table S10). Consequently, the Mediterranean coast of Libya has greater quantities of Cd and Pb^85^. Compared to the Ivra complex in Italy, the concentrations of Co, Mn, and Ni were decreased^86^. The concentrations of Cd, Cr, Cu, Co, Ni, and Pb found along the Moroccan Mediterranean coast were strikingly similar to those found in our investigation^87^. Compared to the beaches of Libya and Malaga Bay, the Egyptian Mediterranean has higher quantities of Cr.

### Possible remedial approaches for PHEs

Because bioaccumulation mechanisms allow PHE ions to enter the food chain and raise concentrations over time, biological systems are harmed by PHEs. Runoff from agricultural operations, industrial processes that release metals, and wastewater from home and commercial use are just a few of the ways that PHEs can enter the aquatic system. Therefore, selecting the right biomass has been highlighted as one of the primary obstacles in the bio-sorption process^88,89^. Previous bioremediation methods have reduced the number of metal ions in wastewater by employing various biomasses. Applications like biosorbents can benefit from the low cost of this biomass^90^. Cell walls, together with other elements, the fungus’s cell walls and other components greatly aid in bio-sorption. Because the dry fungus was grown at different pH values, adsorptive treatment was used to remove the PHEs^91^. Municipal sewage sludge is converted into “bio-solids” by wastewater treatment plants. Bio-solids are becoming increasingly popular as a land application technique, as they recycle nutrients and organic materials while improving the sediment’s structure and quality^92^.

Plants may absorb, accumulate, and occasionally detoxify PHEs through phytoremediation. The phytoremediation process offers an affordable and ecologically beneficial alternative to traditional remediation techniques. Phytoremediation is gaining popularity as a sustainable and environmentally friendly method for removing PHEs from water. Molecular techniques are used to study the absorption, sequestration, translocation, and tolerance of PHEs. This approach is one of the most commonly used methods for reducing the risk of heavy metal contamination in ecosystems or the surrounding environment. Several plant species are utilized, either directly or through genetic modification, to mitigate the adverse effects of agriculture^93^. Numerous external processes (thermal, physical, chemical, and electrical) or inputs (soil hydrogels, ceramics, clays, water, and aeration) work in tandem with plant development^89^.

## Conclusion

A main investigation on the levels of 24 different elements (Li, B, Na, Mg, Al, K, Ca, Ti, Cr, Mn, Fe, Co, Ni, Cu, Zn, Ga, Se, Sr, Ag, Cd, In, Ba, Pb, and Bi) found along Egypt’s western Mediterranean coast is presented in the current communication. To examine element contamination in the sediment samples resulting from numerous operations on Egypt’s west Mediterranean coast, 21 sediment samples were collected from 11 sampling sectors. The average concentration of all the elements was 3402.69 ± 737.29 µg/g. Due to the numerous industrial operations and plants in the vicinity, Sedi Krrir (X) Station exhibits the highest average value in this study. According to *I*_geo_ and EF, this investigation also showed that Cd (2.16 to 4.15) was classified as extremely polluted in all stations examined. With a value of 18.40 for the contamination index (Cd), Sedi Branny station has the highest contamination score. The elements recorded were Fe > Mn > Pb > Cu > Zn, according to CDI readings. The distribution of elements among human tribes was as follows: Cd > Cu > Fe > Mn > Pb > Zn. PEL and ERM levels are lower than the average Cd amounts found in this study. It is also necessary to investigate the anticipated sources of pollution along Egypt’s western Mediterranean coasts. The findings of the correlation study revealed a highly significant positive association between Mn, Fe, Al, and Ni, suggesting that the sources of pollution from these metals may be the same. In Egypt’s Western Mediterranean Sea sediments, toxic metal risks come from both natural (geological, atmospheric) and significant anthropogenic sources, such as mining, industrial/sewage discharge, agricultural runoff (pesticides/fertilizers), and elevated levels of metals like Cd, Pb, Cu, and Zn, which pose moderate to low ecological/health risks. Cd is frequently the highest, requiring improved management and source control. Important sources that affect coastal regions, such as El-Dabaa, El-Manzala, and the Nile Delta, include adjacent industrial zones (Alexandria), agricultural drains, and mining operations.

According to the PCA model’s findings, there are three main categories into which the sources of PHEs pollution may be separated; PC1 is the most prevalent group, explaining 30.61% of the variation. Other toxic metals, such as As and Hg, are also essential to consider in sediment. The buried mercury may be remobilized into the surrounding water because of human activity or physical, chemical, or biological processes (such as hydrodynamic fluxes, bioturbation, molecular diffusion, and chemical transformation). The reactivity (i.e., the conversion of inorganic Hg(II) to MeHg), transport, and exposure of living organisms to mercury are all significantly impacted by mercury speciation in the water column and sediments. Additionally, the availability of methylating bacteria and their activity are influenced by geological circumstances. The same is true for As; thus, we recommend studying their speciation in sediment for further investigation.

## Supplementary Information

Below is the link to the electronic supplementary material.

Supplementary Material 1

## Data Availability

This published article and its supplementary information files contain all the data produced or analyzed throughout this study.

## References

[1] Zhao, Y. et al. Study of heavy metal pollution, ecological risk and source apportionment in the surface water and sediments of the Jiangsu coastal region, China: A case study of the Sheyang estuary. *Mar. Poll. Bull.* **137**, 601–609 (2018). 10.1016/j.marpolbul.2018.10.044 30503473

[2] Chen, J. et al. Study on Spatial distribution, potential sources and ecological risk of heavy metals in the surface water and sediments at Shanghai Port, China. *Mar. Poll. Bull.* **181**, 113923 (2022). 10.1016/j.marpolbul.2022.113923 35843161

[3] Hassaan, M. A., Eldeeb, T. & Nemr, E. A. Pesticides removal techniques from aquatic environment. Book entitled: Pesticides in the Natural Environment. Chapter 19, 483–516 https://doi.org/B978-0-323-90489-6.00019-7 (2022).

[4] Shetaia, S. A., Khatita, A. M. A., Abdelhafez, N. A., Shaker, I. M. & Kafrawy, E. Human-induced sediment degradation of burullus lagoon, nile Delta, egypt: heavy metals pollution status and potential ecological risk. *Mar. Poll. Bull.* **178**, 113566 (2022). 10.1016/j.marpolbul.2022.113566 35366554

[5] Magnusson, K. et al. Risk assessment of bilge water discharges in two Baltic shipping lanes. *Mar. Poll. Bull.* **126**, 575–584 (2018). 10.1016/j.marpolbul.2017.09.035 28982478

[6] Ytreberg, E. et al. Metal and PAH loads from ships and boats, relative other sources, in the Baltic sea. *Mar. Poll. Bull.* **182**, 113904 (2022). 10.1016/j.marpolbul.2022.113904 35878478

[7] Yu, S. et al. Surface sediment quality relative to Port activities: A contaminant-spectrum assessment. *Sci. Total Environ.* **596**, 342–350 (2017). 28441574 10.1016/j.scitotenv.2017.04.076

[8] Hassaan, M. A. & El-Rayis, O. Calculation and solutions for heavy metals pollution load from Umum and Qalaa drains to lake Mariut, Egypt. *Indian J. Geo-Mar. Sci.* **47** (4), 1460–1467 (2018).

[9] Elgendy, A. R. et al. Evaluation of some leachable heavy metals in the seafloor sediments of the two navigation harbours El Zaitiya and Adabiya, Gulf of Suez, Egypt. *Egypt. J. Aqu Biol. Fisher*. **22** (4), 77–92 (2018).

[10] Cai, P. et al. Distribution, risk assessment, and quantitative source apportionment of heavy metals in surface sediments from the shelf of the Northern South China sea. *Mar. Poll. Bull.* **187**, 114589 (2023). 10.1016/j.marpolbul.2023.114589 36646001

[11] Vetrimurugan, E. et al. Comprehensive study on metal contents and their ecological risks in beach sediments of KwaZulu-Natal province, South Africa. *Mar. Poll. Bull.* **149**, 110555 (2019). 10.1016/j.marpolbul.2019.110555 31542597

[12] Kumar, S. B., Padhi, R. K., Mohanty, A. K. & Satpathy, K. K. Distribution and ecological-and health-risk assessment of heavy metals in the seawater of the Southeast Coast of India. *Mar. Poll. Bull.* **161**, 111712 (2020). 10.1016/j.marpolbul.2020.111712 33065393

[13] Varol, M., Ustaoğlu, F. & Tokatlı, C. Ecological risks and controlling factors of trace elements in sediments of dam lakes in the black sea region (Turkey). *Environ. Res.* **205**, 112478 (2022). 34863685 10.1016/j.envres.2021.112478

[14] Da Le, N. et al. Evaluation of heavy metal contamination in the coastal aquaculture zone of the red river delta (Vietnam). *Chemosphere* **303**, 134952 (2022). 35595107 10.1016/j.chemosphere.2022.134952

[15] Xiao, H. et al. Source-specific ecological risk assessment and quantitative source apportionment of heavy metals in surface sediments of Pearl river Estuary, China. *Mar. Poll. Bull.* **179**, 113726 (2022). 10.1016/j.marpolbul.2022.113726 35567962

[16] Deng, L. et al. Spatial and Temporal variation of dissolved heavy metals in the Lijiang River, china: implication of rainstorm on drinking water quality. *Environ. Sci. Poll. Res.* **28**, 68475–68486 (2021). 10.1007/s11356-021-15383-3 34275078

[17] Ashayeri, S. Y., Keshavarzi, B., Moore, F., Ahmadi, A. & Hooda, P. S. Risk assessment, geochemical speciation, and source apportionment of heavy metals in sediments of an urban river draining into a coastal wetland. *Mar. Poll. Bull.* **186**, 114389 (2023). 10.1016/j.marpolbul.2022.114389 36462421

[18] Kalnejais, L. H., Martin, W. R., Signell, R. P. & Bothner, M. H. Role of sediment resuspension in the remobilization of particulate-phase metals from coastal sediments. *Environ. Sci. Tech.* **41** (7), 2282–2288. 10.1021/es061770z (2007). 10.1021/es061770z 17438776

[19] Liu, B., Wang, J., Xu, M., Zhao, L. & Wang, Z. Spatial distribution, source apportionment and ecological risk assessment of heavy metals in the sediments of Haizhou Bay National ocean park, China. *Mar. Poll. Bull.* **149**, 110651 (2019).

[20] Siddique, M. A. M. et al. Assessment of heavy metal contamination in the surficial sediments from the lower Meghna river estuary, Noakhali coast, Bangladesh. *Inter J. Sed Res.* **36** (3), 384–391 (2021).

[21] Linnik, P. M. & Zubenko, I. B. Role of bottom sediments in the secondary pollution of aquatic environments by heavy-metal compounds. *Lakes Reservoirs: Res. Manage.* **5** (1), 11–21 (2000).

[22] El Nemr, A. M., El Sikaily, A. & Khaled, A. Total and leachable heavy metals in muddy and sandy sediments of the Egyptian Coast along the mediterranean sea. *Environ. Monit. Assess.* **129**, 151–168. 10.1007/s10661-006-9349-8 (2007). 17057978 10.1007/s10661-006-9349-8

[23] El Rayis, O. A., Hassaan, M. A. & Hemada, E. I. Suitability of lake Mariut drainage system (Qalaa and Umum drains waters) for water reuse. *Blue Biotechnol. J.* **3** (2), 265 (2014).

[24] Zhao, Z. et al. Ecological risk assessment of trace metals in sediments and their effect on benthic organisms from the South Coast of Zhejiang province, China. *Mar. Poll. Bull.* **187**, 114529 (2023). 10.1016/j.marpolbul.2022.114529 36608476

[25] Rainbow, P. S. Trace metal bioaccumulation: Models, metabolic availability and toxicity. *Environ. Inter*. **33** (4), 576–582 (2007). 10.1016/j.envint.2006.05.007 16814385

[26] Amato, E. D. et al. Assessing the effects of bioturbation on metal bioavailability in contaminated sediments by diffusive gradients in thin films (DGT). *Environ. Sci. Tech.* **50** (6), 3055–3064 (2016). 10.1021/acs.est.5b04995 26848961

[27] El-Gammal, M., Shata, M., Hamouda, A. & El-Gharabawy, S. Assessment of heavy metals concentration in mediterranean surficial sediments in front of Damietta promontory, Egypt. *J. Environ. Sci.* **42** (3), 417–432 (2013).

[28] Yin, K., Wang, Q., Lv, M. & Chen, L. Microorganism remediation strategies towards heavy metals. *Chem. Eng. J.* **360**, 1553–1563 (2019).

[29] Sigué, C. et al. Assessment of contamination, sources and health risks of potentially hazardous elements in surface sediments of Dibang, Cameroon. *Discov Environ.* **3**, 165. 10.1007/s44274-025-00363-y (2025).

[30] Osfor, M. M. H., El-Dessouky, S. A., El‐Sayed, A. & Higazy, R. A. Relationship between environmental pollution in Manzala lake and health profile of fishermen. *Food/Nahrung* **42** (01), 42–45 (1998). 9584278 10.1002/(sici)1521-3803(199802)42:01<42::aid-food42>3.3.co;2-x

[31] Soliman, A. S. et al. Geographical clustering of pancreatic cancers in the Northeast nile delta region of Egypt. *Arch. Environ. Contam. Tox*. **51**, 142–148 (2006). 10.1007/s00244-005-0154-0 16453066

[32] Alomary, A. A. & Belhadj, S. Determination of heavy metals (Cd, Cr, Cu, Fe, Ni, Pb, Zn) by ICP-OES and their speciation in Algerian mediterranean sea sediments after a five-stage sequential extraction procedure. *Environ. Monit. Assess.* **135**, 265–280 (2007). 17342430 10.1007/s10661-007-9648-8

[33] Simou, A. et al. Assessing ecological and health risks of potentially toxic elements in marine and beach sediments of Tangier Bay, Southwestern mediterranean Sea, marine pollution bulletin 209 (Part B), 117234 (2024a). 10.1016/j.marpolbul.2024.117234 10.1016/j.marpolbul.2024.117234 39522119

[34] Simou, A. et al. Assessment of ecological risk and metal contamination caused by the Lihoud river emissary along the Bay of Tangier Littoral in Morocco (Southwestern mediterranean Sea). *Environ. Qual. Manage.* **34** (Issue 2), e22269. 10.1002/tqem.22269 (2024b).

[35] Afahnwie, N. A., Embui, V. F., Yiika, L. P., Djibril, K. N. G. & Kehding, F. B. Preliminary stream sediment geochemical exploration for base metals and other elements in terms of source apportionment and contamination status of Manjo and environs, Cameroon. *Discov Chem.* **2**, 93. 10.1007/s44371-025-00183-2 (2025).

[36] El Nemr, A. & Impact Monitoring and management of environmental pollution. in Pollution Science, Technology and Abatement Series. 638 (Nova Science Publishers, Inc., 2010).

[37] Harikumar, P. S. & Nasir, U. P. Ecotoxicological impact assessment of heavy metals in core sediments of a tropical estuary. *Ecotoxicol. Environ. Saf.* **73** (7), 1742–1747 (2010). 20817297 10.1016/j.ecoenv.2010.08.022

[38] El-Sikaily, A., Khaled, A. & El Nemr, A. *Heavy Metal Contaminations in Mediterranean sediments. Impact, Monitoring and Management of Environmental Pollution. Book Edited by Ahmed El Nemr* 223–262 (Nova Science Publishers, Inc. Hauppauge New York, 2011).

[39] Pekey, H. The distribution and sources of heavy metals in Izmit Bay surface sediments affected by a polluted stream. *Mar. Poll. Bull.* **52** (10), 1197–1208 (2006). 10.1016/j.marpolbul.2006.02.012 16580024

[40] Khaled, A., El Nemr, A. & El Sikaily, A. Leachable and total heavy metals contamination in mediterranean surface sediment along the Egyptian Coast. *Blue Biotech. J.* **1** (1), 113–140 (2012).

[41] Hassaan, M. A. & El Nemr, A. Classification and identification of different minerals in the mediterranean sediments using PSA, FTIR, and XRD techniques. *Mar. Poll. Bull.* **173**, 113070. 10.1016/j.marpolbul.2021.113070 (2021). 10.1016/j.marpolbul.2021.113070 34678547

[42] Tokatlı, C. et al. Spatial-temporal variations of inorganic contaminants and associated risks for sediment of felent stream basin flowing along with silver mines in the Midwestern Türkiye. *Soil. Sediment. Contamination: Int. J.* **34** (7), 1853–1870. 10.1080/15320383.2025.2464153 (2025).

[43] Öncü, T., Yazman, M. M., Ustaoğlu, F., Hristova, E. & Yuksel, B. Source dynamics and environmental risk of street dust as a vector of human exposure to potentially toxic elements in Istanbul, Türkiye. *Sci. Rep.* **15**, 30550. 10.1038/s41598-025-11472-2 (2025). 40835637 10.1038/s41598-025-11472-2 PMC12368213

[44] Varol, M., Ustaoğlu, F. & Tokatlı, C. Metal pollution, eco-health risks and source apportionment in coastal sediments of Samsun, Türkiye: A receiving zone for the Kızılırmak and Yeşilırmak rivers. *Environ. Res.* **282**, 122113. 10.1016/j.envres.2025.122113 (2025). 40499644 10.1016/j.envres.2025.122113

[45] Badawi, A. et al. Severity gradient of anthropogenic activities along the Egyptian Western mediterranean coast, utilizing benthic foraminifera as bio-indicators. *Egypt. J. Aquat. Res.* **48**, 45–52. 10.1016/j.ejar.2022.01.006 (2022).

[46] Okbah, M. A., Ibrahim, A. M. A. & Gamal, M. N. M. Environmental monitoring of linear alkylbenzene sulfonates and physicochemical characteristics of seawater in El- Mex Bay (Alexandria, Egypt). *Environ. Monit. Assess.* **185**, 3103–3115. 10.1007/S10661-012-2776-9 (2013). 22851193 10.1007/s10661-012-2776-9 PMC3586065

[47] Khaled, A., Abdel-Halim, A., El-Sherif, Z. & Mohamed, L. Health risk assessment of some heavy metals in water and sediment at Marsa-Matrouh, mediterranean Sea, Egypt. *J. Environ. Prot.* **8** (1), 74–97. 10.4236/jep.2017.81007 (2017).

[48] Badawi, A. & Magdy, S. M. Evaluation of the pollution extent of heavy metals in the sediment of the nile Delta, mediterranean Coast, Egypt. *Egypt J. Aquat. Res.* **49** (2), 221–228. 10.1016/j.ejar.2023.01.002 (2023).

[49] USEPA. Method 3051A (SW-846): Microwave assisted acid digestion of sediments, sludges, and oils (Revision 1). (2007). Available at: http://www.jonesenv.com/PDF/3051a.pdf

[50] Muller, G. Index of geoaccumulation in sediments of the rhine river. *Geojournal* **2**, 108–118 (1969).

[51] Salomons, W. & Förstner, U. Metals in the Hydrocycle. Springer Science & Business Media (1984). 10.1007/978-3-642-69325-0

[52] Buat-Menard, P. & Chesselet, R. Variable influence of the atmospheric flux on the trace metal chemistry of oceanic suspended matter. *Earth Planet. Sci. Lett.* **42**, 398–411 (1979).

[53] Tomlinson, D. L., Wilson, J. G., Harris, C. R. & Jeffrey, D. W. Problems in the assessment of heavy-metal levels in estuaries and the formation of a pollution index. *Helgoländer Meeresuntersuchungen*. **33**, 566–575 (1980).

[54] Backman, B., Bodiš, D., Lahermo, P., Rapant, S. & Tarvainen, T. Application of a groundwater contamination index in Finland and Slovakia. *Environ. Geol.* **36** (1), 55–64. 10.1007/s002540050320 (1998). https://link.springer.com/article/

[55] Avvari, L., Basuri, C. K., Chari, N. V. H. K., Tirukkovalluri, S. R. & Gollapalli, N. R. Assessment of heavy metal distribution in seawater of Kakinada Bay, a tropical mangrove-rich coastal environment. *Mar. Poll. Bull.* **181**, 113877 (2022). 10.1016/j.marpolbul.2022.113877 35777325

[56] Kim, E., Little, J. C. & Chiu, N. Estimating exposure to chemical contaminants in drinking water. *Environ. Sci. Tech.* **38** (6), 1799–1806 (2004). 10.1021/es026300t 15074692

[57] Bhagat, C., Kumar, M., Mahlknecht, J., Hdeib, R. & Mohapatra, P. K. Seawater intrusion decreases the metal toxicity but increases the ecological risk and degree of treatment for coastal groundwater: an Indian perspective. *Environ. Poll.* **310**, 119771 (2022). 10.1016/j.envpol.2022.119771 35863708

[58] Shetaia, S. A. et al. Assessment of heavy metals contamination of sediments and surface waters of bitter Lake, Suez Canal, egypt: ecological risks and human health. *Mar. Poll. Bull.* **192**, 115096 (2023). 10.1016/j.marpolbul.2023.115096 37271076

[59] ATSDR [Agency for Toxic Substances and Disease Registry]. *Exposure Dose Guidance for Soil/Sediment Dermal Absorption*. Atlanta, GA: U.S. Department of Health and Human Services, Public Health Service, July 2023. (2023).

[60] Shetty, B. R., Pai, B. J., Salmataj, S. A. & Naik, N. Assessment of carcinogenic and non-carcinogenic risk indices of heavy metal exposure in different age groups using Monte Carlo simulation approach. *Sci. Rep.* **14** (1), 30319. 10.1038/s41598-024-81109-3 (2024). 39638837 10.1038/s41598-024-81109-3 PMC11621557

[61] USEPA. Reference dose (RfD): description and use in health risk assessments. Background document 1A: Integrated Risk Information System (IRIS) Washington (DC). (1993).

[62] Chow, T. E., Gaines, K. F., Hodgson, M. E. & Wilson, M. D. Habitat and exposure modelling for ecological risk assessment: A case study for the raccoon on the Savannah river site. *Ecol. Mod.* **1891** (2), 151–167 (2005).

[63] Abdullah, M. I. C., Sah, A. S. R. M. & Haris, H. G. Index and enrichment factor of arsenic in surface sediment of bukit merah reservoir, Malaysia. Trop Life Sci Res. 31(3):109–125 (2020). 10.21315/tlsr2020.31.3.8 10.21315/tlsr2020.31.3.8 PMC7652249 33214859

[64] Birth, G. A scheme for assessing human impacts on coastal aquatic environments using sediments. *Coastal Gis* **14** (2003).

[65] Emenike, P. C. et al. An integrated assessment of land-use change impact, seasonal variation of pollution indices and human health risk of selected toxic elements in sediments of river Atuwara. *Nigeria Environ. Poll.* **265**, 114795 (2020). 10.1016/j.envpol.2020.114795 32531623

[66] Bakan, G. & Ozkoc, H. B. An ecological risk assessment of the impact of heavy metals in surface sediments on biota from the mid-Black sea Coast of Turkey. *Int. J. Environ. Stud.* **64** (I), 45–57 (2007).

[67] MacDonald, D. D., Ingersoll, C. G. & Berger, T. A. Development and evaluation of consensus-based sediment quality guidelines for freshwater ecosystems. *Archives Environ Contam. Toxicol*. **39**, 20–31 (2000). 10.1007/s002440010075 10790498

[68] Perin, G. et al. Heavy metal pollution in central Venice lagoon bottom sediments: Evaluation of the metal bioavailability by geochemical speciation procedure. *Environ. Technol.* **18**, 593–604 (1997).

[69] Eleryan, A. et al. Copper (II) ion removal by chemically and physically modified sawdust Biochar. *Biomass Convers. Biorefinery* 1–38 (2022).

[70] El-Nemr, M. A. et al. Adsorption of Cr6 + ion using activated *Pisum sativum* peels-triethylenetetramine. *Environ. Sci. Pollut. Res.* **29** (60), 91036–91060 (2022). 10.1007/s11356-022-21957-6 PMC9722890 35881295

[71] Said, T. O., Ragab, S., El Sikaily, A., Hassaan, M. A. & Nemr, E. Distribution, composition and risk assessment of hydrocarbon residue in surficial sediments of El-Dakhla, El-Kharga and El-Farafra oases. *Egypt. Sci. Rep.* **13** (1), 18871 (2023). 37914771 10.1038/s41598-023-46133-9 PMC10620400

[72] Ali, R. M. et al. Towards potential removal of malachite green from wastewater: Adsorption process optimization and prediction. In *Materials Science Forum* 1008, 213–221 (Trans Tech Publications Ltd, 2020).

[73] Hassaan, M. A., El Nemr, A., El Sikaily, A. & Ragab, S. n-Alkanes and PAHs baseline distributions and sources in the sediments of the nile delta Coast of the southeastern mediterranean. *Mar. Pollut. Bull.* **194**, 115262 (2023). 37467685 10.1016/j.marpolbul.2023.115262

[74] Hassaan, M. A., El-Rayis, O., Hemada, E. & Nemr, E. Assessment of potentially toxic elements in water and sediments in the drainage network of lake Mariout, Egypt. *SN Appl. Sci.* **4** (8), 239 (2022).

[75] Hassaan, M. A., Elkatory, M. R., Ragab, S. & Nemr, E. Polychlorinated biphenyls (PCBs) and organochlorine pesticides (OCPs) in water-sediment system of Southern mediterranean: Concentration, source and ecological risk assessment. *Mar. Pollut. Bull.* **196**, 115692 (2023). 37871457 10.1016/j.marpolbul.2023.115692

[76] Hassaan, M. A., Ragab, S., Sikaily, A. E. & Nemr, A. E. Sources of hydrocarbons and their risk assessment in seawater and sediment samples collected from the nile delta Coast of the mediterranean sea. *Sci. Rep.* **14** (1), 5082 (2024). 38429376 10.1038/s41598-024-55339-4 PMC10907701

[77] Elgendy, A., Hassaan, A., Soliman, M. A., Dar, A. & F. and The impact of maritime activities on the leachable heavy metals in the seafloor sediments of Port Tawfiq and Tersana harbours, Gulf of Suez, Egypt. *Egypt. J. Aquat. Biology Fisheries*. **22** (5), 523–536 (2019).

[78] Miletić, A., Lučić, M. & Onjia, A. Exposure factors in health risk assessment of heavy metal(loid)s in soil and sediment. Metals 13, 1266. (2023). 10.3390/met13071266

[79] Qiao, M. et al. Characterization of soil heavy metal contamination and potential health risk in metropolitan region of Northern China. *Environ. Monit. Assess.* **172**, 353–365 (2011). 20135216 10.1007/s10661-010-1339-1

[80] Spurgeon, D. J. et al. Geographical and pedological drivers of distribution and risks to soil fauna of seven metals (Cd, Cu, Cr, Ni, Pb, V and Zn) in British soils. *Environ. Poll.* **153** (2), 273–283 (2008). 10.1016/j.envpol.2007.08.027 17950507

[81] Kachoueiyan, F., Karbassi, A., Nasrabadi, T., Rashidiyan, M. & De-la-Torre, G. E. Speciation characteristics, ecological risk assessment, and source apportionment of heavy metals in the surface sediments of the Gomishan wetland. *Mar. Pollut. Bull.* **198**, 115835. 10.1016/j.marpolbul.2023.115835 (2024). 38039575 10.1016/j.marpolbul.2023.115835

[82] Csomós, E., Héberger, K. & Simon-Sarkadi, L. Principal component analysis of biogenic amines and polyphenols in Hungarian wines. *J. Agr Food Chem.* **50** (13), 3768–3774 (2002). 12059158 10.1021/jf011699a

[83] Pardo, R., Vega, M., Debán, L., Cazurro, C. & Carretero, C. Modelling of chemical fractionation patterns of metals in soils by two-way and three-way principal component analysis. *Anal. Chim. Acta*. **606** (1), 26–36 (2008). 18068767 10.1016/j.aca.2007.11.004

[84] Van Wijngaarden, R. P. A., Van Den Brink, P. J., Oude Voshaar, J. H. & Leeuwangh, P. Ordination techniques for analysing response of biological communities to toxic stress in experimental ecosystems. *Ecotoxicology* **4** (1), 61–77 (1995). 24197549 10.1007/BF00350650

[85] Soliman, N. F., Nasr, S. M. & Okbah, M. A. Potential ecological risk of heavy metals in sediments from the mediterranean coast, Egypt. *J. Environ. Health Sci. Eng.* **13**, 1–12 (2015). 26457189 10.1186/s40201-015-0223-x PMC4600254

[86] Hartmann, G. & Wedepohl, K. H. The composition of peridotite tectonites from the Ivrea Complex, Northern italy: Residues from melt extraction. *Geochim. Cosmochim. Acta*. **57**, 1761–1782. 10.1016/0016-7037(93)90112-A (1993).

[87] Omar, M. B. et al. Distribution of heavy metals in marine sediments of Tetouan Coast (North of Morocco): natural and anthropogenic sources. *Environ. Earth Sci.* **74**, 4171–4185 (2015).

[88] Wang, J. & Chen, C. Biosorbents for heavy metals removal and their future. *Biotechnol. Adv.* **27** (2), 195–226 (2009). 19103274 10.1016/j.biotechadv.2008.11.002

[89] Sharma, A. K. et al. A systematic review on assessment of heavy metals toxicity in freshwater fish species: Current scenario and remedial approaches. *J. Geochem. Explor.* **262**, 107472. 10.1016/j.gexplo.2024.107472 (2024).

[90] Maurya, N. S., Mittal, A. K., Cornel, P. & Rother, E. Biosorption of dyes using dead macro fungi: Effect of 725 dye structure, ionic strength and pH. *Bioresour Technol.* **97** (3), 512–521 (2006). 16216733 10.1016/j.biortech.2005.02.045

[91] Lalmi, B. K. E., Sahraoui, B. & el Houda, Anfif, C. Removal of lead from polluted waters using ionexchange resin with Ca (NO_3_)_2_ for elution. *Hydrometallurgy* 287–293, (2018).

[92] Das, A., Mandal, B., Sarkar, J. & Chaudhuri, S. Bioaccumulation of heavy metals in some commercial fishes and crabs of the Gulf of Cambay. *India Sci* **92** (11), (2007).

[93] Schück, M. & Greger, M. Plant traits related to the heavy metal removal capacities of wetland 773 plants. *Int. J. Phytoremediat*. **22** (4), 427–435 (2020). 10.1080/15226514.2019.1669529 31594381

