# Supplementary material for: Ecological and human health risks of metal contamination in sediments along Egypt Western Mediterranean coast
Source: Sci Rep. 2026 Mar 11;16:8725. doi: 10.1038/s41598-026-39462-y (PMC12979678; doi:10.1038/s41598-026-39462-y)
Supplement: Supplementary file 1 — Supplementary Material 1 [file 41598_2026_39462_MOESM1_ESM.docx]

**Supplementary data**

**Ecological and Human Health Risks of Metal Contamination in Sediments along Egypt Western Mediterranean Coast**

Mohamed A. Hassaan^1^, Amr G. Dardeer^2^, Tarek O. Said^1^, Mahmoud M. El-Mezayen^1^, Ahmed El Nemr^1*^

^1^ Environment Division, National Institute of Oceanography and Fisheries (NIOF), Kayet Bey, Elanfoushy, Alexandria, Egypt

^2^ Administration of Environmental Monitoring - Ministry of Health and Population, Alexandria, Egypt.

Email: [mhss95@mail.com](mailto:mhss95@mail.com) (M.A. Hassaan); [amrbiochemistry@gmail.com](mailto:amrbiochemistry@gmail.com) (A.G. Dardeer); [tareksaideg@yahoo.co.uk](mailto:tareksaideg@yahoo.co.uk) (T.O. Said); [m.elmezayen@gmail.com](mailto:m.elmezayen@gmail.com) (M.M. El-Mezayen)

*Corresponding author Email: [ahmedmoustafaelnemr@yahoo.com](mailto:ahmedmoustafaelnemr@yahoo.com)

**Table S1** Shows the stations locations with their coordinates.

| **Stations** | **Depths (m)** | **Labels** | **Latitudes** | **Longitudes** |
| --- | --- | --- | --- | --- |
| **Western section** | | | | |
| Salloum | 45 | Ia | 31.69500000 N | 25.50194444 E |
|  | 150 | Ib | 31.73666667 N | 25.285 E |
|  | 350 | Ic | 31.92916667 N | 25.33416667 E |
| East of Salloum | 43 | II | 31.74111111 N | 25.70916667 E |
| Sedi Branny | 48 | IIIa | 31.72777778 N | 26.33833333 E |
|  | 105 | IIIb | 31.72388889 N | 26.24111111 E |
|  | 481 | IIIc | 31.94444444 N | 26.39388889 E |
| El Negalia | 87 | IV | 31.61194444 N | 26.69277778 E |
| Marsa Matrouh | 117 | V | 31.50111111 N | 27.58916667 E |
| Rome Flag | 60 | VI | 31.36666667 N | 28.28722222 E |
| Ras El Hikma | 65 | VIIa | 31.36805556 N | 28.42555556 E |
|  | 131 | VIIb | 31.23777778 N | 28.42722222 E |
| El Dabaa | 45 | VIIIa | 31.11916667 N | 28.83277778 E |
|  | 60 | VIIIb | 31.11194444 N | 29.03 E |
|  | 230 | VIIIc | 31.07833333 N | 28.8675 E |
| El Alamein | 51 | IXa | 32.07138889 N | 29.43166667 E |
|  | 170 | IXb | 31.03361111 N | 29.35944444 E |
|  | 305 | IXc | 31.11083333 N | 29.455 E |
| **Central section** | | | | |
| Sedi krrir | 250 | X | 31.56 N | 29.76805556 E |
| El Mex | 50 | XIa | 31.52 N | 30.03361111 E |
|  | 275 | XIb | 31.58583333 N | 29.84583333 E |

**Table S2**. Analytical results of certified and obtained values (ppm) of elements in standard reference materials SRM 2702 (n = 21). Percentage (%) was used as the measurement unit of the major elements (Na, Mg, Al, K, Ca, Ti and Fe).

|  | **Element** |  | **SRM 2702** |  |
| --- | --- | --- | --- | --- |
|  |  | Certified | Measured | Recovery (%) |
| 1 | Li | 78.2 | 77.2 | 98.7 |
| 3 | Na | 0.681* | 0.685 | 100.6 |
| 4 | Mg | 0.990* | 0.999 | 100.9 |
| 5 | Al | 8.41* | 8.30 | 98.7 |
| 6 | K | 2.054* | 2.001 | 97.4 |
| 7 | Ca | 0.343* | 0.335 | 97.7 |
| 8 | Ti | 0.884* | 0.879 | 99.4 |
| 9 | Cr | 352 | 347 | 98.6 |
| 10 | Mn | 1757 | 1732 | 98.6 |
| 11 | Fe | 7.91* | 7.70 | 97.3 |
| 12 | Co | 27.76 | 27.45 | 98.9 |
| 13 | Ni | 75.4 | 74.7 | 99.1 |
| 14 | Cu | 117.7 | 114.5 | 97.6 |
| 15 | Zn | 485.3 | 478.5 | 98.6 |
| 16 | Ga | 24.3 | 23.6 | 97.1 |
| 17 | Se | 4.95 | 4.84 | 97.7 |
| 18 | Sr | 119.7 | 118.5 | 99.0 |
| 19 | Ag | 0.622 | 0.619 | 99.7 |
| 20 | Cd | 0.817 | 0.814 | 99.6 |
| 22 | Ba | 397.4 | 396.1 | 99.7 |
| 23 | Pb | 132.8 | 131.5 | 99.0 |

**Table S3** Chronic Daily Intake (CDI) for dermal absorption in different elements

| **Site** | **Al** | **Ti** | **Cr** | **Mn** | **Fe** | **Co** | **Ni** | **Cu** | **Zn** | **Cd** | **Pb** |
| --- | --- | --- | --- | --- | --- | --- | --- | --- | --- | --- | --- |
| **Ia** | 3.39E-02 | 2.15E-02 | 5.62E-04 | 2.30E-04 | 7.66E-02 | 5.64E-04 | 2.41E-04 | 4.05E-03 | 2.05E-03 | 4.62E-05 | 7.33E-04 |
| **Ib** | 4.85E-02 | 4.63E-02 | 3.58E-04 | 2.76E-04 | 9.06E-02 | 5.76E-04 | 2.16E-04 | 2.72E-03 | 1.77E-03 | 7.80E-05 | 8.45E-04 |
| **Ic** | 5.70E-02 | 5.45E-02 | 3.73E-04 | 5.66E-04 | 1.44E-01 | 7.32E-04 | 2.90E-04 | 4.69E-03 | 6.10E-03 | 1.19E-03 | 7.84E-04 |
| **II** | 1.65E-02 | 2.00E-01 | 3.15E-05 | 6.07E-05 | 2.26E-02 | 4.07E-04 | 1.65E-04 | 7.96E-04 | 9.70E-04 | 2.12E-05 | 3.40E-04 |
| **IIIa** | 1.15E-02 | 2.55E-01 | 3.85E-05 | 6.55E-05 | 7.10E-02 | 3.16E-04 | 1.55E-04 | 3.27E-04 | 1.91E-03 | 1.51E-05 | 7.98E-05 |
| **IIIb** | 4.24E-02 | 2.03E-01 | 4.56E-05 | 4.67E-04 | 9.73E-02 | 8.24E-04 | 3.02E-04 | 2.40E-03 | 2.22E-03 | 4.65E-05 | 8.68E-04 |
| **IIIc** | 8.23E-02 | 1.78E-01 | 6.19E-05 | 3.60E-04 | 6.34E-02 | 1.05E-03 | 3.59E-04 | 1.68E-03 | 1.64E-03 | 3.36E-05 | 1.06E-03 |
| **IV** | 5.92E-02 | 1.47E-01 | 4.82E-05 | 1.47E-04 | 4.88E-02 | 8.32E-04 | 2.67E-04 | 1.37E-03 | 1.16E-03 | 2.18E-05 | 9.66E-04 |
| **V** | 7.09E-02 | 1.48E-01 | 5.97E-05 | 5.73E-04 | 2.74E-02 | 1.08E-03 | 3.28E-04 | 2.29E-03 | 3.05E-03 | 6.80E-05 | 3.95E-04 |
| **VI** | 4.93E-02 | 1.64E-01 | 4.37E-05 | 5.34E-04 | 3.98E-02 | 6.87E-04 | 1.89E-04 | 1.66E-03 | 1.86E-03 | 6.04E-05 | 7.19E-04 |
| **VIIa** | 2.68E-02 | 2.13E-01 | 2.12E-05 | 7.17E-05 | 2.49E-02 | 2.14E-03 | 1.15E-04 | 1.25E-03 | 1.97E-03 | 3.55E-05 | 8.30E-03 |
| **VIIb** | 3.11E-02 | 1.56E-01 | 2.52E-05 | 2.51E-04 | 2.03E-02 | 1.27E-03 | 1.55E-04 | 1.52E-03 | 1.30E-03 | 4.94E-05 | 8.50E-05 |
| **VIIIa** | 2.83E-02 | 2.48E-01 | 1.95E-05 | 4.13E-05 | 2.86E-02 | 9.97E-04 | 1.27E-04 | 1.72E-03 | 1.03E-03 | 4.46E-05 | 5.77E-04 |
| **VIIIb** | 2.71E-02 | 2.20E-01 | 2.37E-05 | 6.82E-05 | 3.25E-02 | 1.00E-03 | 1.27E-04 | 1.56E-03 | 7.23E-04 | 3.06E-05 | 7.26E-04 |
| **VIIIc** | 4.35E-02 | 2.01E-01 | 2.73E-05 | 1.04E-04 | 3.47E-02 | 1.76E-03 | 1.66E-04 | 2.12E-03 | 1.13E-03 | 4.79E-05 | 7.86E-04 |
| **IXa** | 3.53E-02 | 2.47E-01 | 2.13E-05 | 5.43E-05 | 3.02E-02 | 7.71E-04 | 1.29E-04 | 1.07E-03 | 9.14E-04 | 3.97E-05 | 3.55E-03 |
| **IXb** | 2.40E-02 | 1.96E-01 | 2.09E-05 | 1.26E-04 | 3.16E-02 | 1.28E-03 | 1.26E-04 | 1.02E-03 | 6.59E-04 | 2.95E-05 | 5.40E-04 |
| **IXc** | 6.99E-02 | 2.08E-01 | 3.56E-05 | 1.75E-04 | 6.42E-02 | 2.35E-03 | 1.74E-04 | 1.46E-03 | 5.35E-04 | 3.22E-05 | 1.01E-03 |
| **X** | 1.04E-01 | 2.19E-01 | 4.67E-05 | 2.69E-04 | 9.53E-02 | 3.08E-03 | 2.17E-04 | 1.91E-03 | 1.21E-03 | 3.89E-05 | 1.96E-03 |
| **XIa** | 4.51E-02 | 2.11E-01 | 2.64E-05 | 9.71E-05 | 4.60E-02 | 1.68E-03 | 1.75E-04 | 2.20E-03 | 8.92E-04 | 4.38E-05 | 1.14E-03 |
| **XIb** | 1.74E-01 | 1.52E-01 | 5.47E-05 | 1.10E-03 | 1.46E-01 | 2.22E-03 | 2.98E-04 | 1.82E-03 | 6.27E-04 | 2.98E-05 | 7.26E-04 |

**Table S4** Chronic Daily Intake (CDI) for dermal absorption in females

| **Site** | **Al** | **Ti** | **Cr** | **Mn** | **Fe** | **Co** | **Ni** | **Cu** | **Zn** | **Cd** | **Pb** |
| --- | --- | --- | --- | --- | --- | --- | --- | --- | --- | --- | --- |
| **Ia** | 3.02E-02 | 1.91E-02 | 4.99E-04 | 2.04E-04 | 6.81E-02 | 5.01E-04 | 2.14E-04 | 3.60E-03 | 1.82E-03 | 4.11E-05 | 6.51E-04 |
| **Ib** | 4.31E-02 | 4.12E-02 | 3.18E-04 | 2.45E-04 | 8.06E-02 | 5.12E-04 | 1.92E-04 | 2.42E-03 | 1.57E-03 | 6.93E-05 | 7.51E-04 |
| **Ic** | 5.07E-02 | 4.84E-02 | 3.31E-04 | 5.03E-04 | 1.28E-01 | 6.51E-04 | 2.58E-04 | 4.16E-03 | 5.42E-03 | 1.06E-03 | 6.97E-04 |
| **II** | 1.47E-02 | 1.77E-01 | 2.80E-05 | 5.40E-05 | 2.01E-02 | 3.62E-04 | 1.47E-04 | 7.08E-04 | 8.63E-04 | 1.88E-05 | 3.02E-04 |
| **IIIa** | 1.03E-02 | 2.27E-01 | 3.42E-05 | 5.82E-05 | 6.31E-02 | 2.81E-04 | 1.38E-04 | 2.91E-04 | 1.70E-03 | 1.34E-05 | 7.09E-05 |
| **IIIb** | 3.77E-02 | 1.80E-01 | 4.06E-05 | 4.15E-04 | 8.65E-02 | 7.32E-04 | 2.69E-04 | 2.14E-03 | 1.97E-03 | 4.14E-05 | 7.72E-04 |
| **IIIc** | 7.31E-02 | 1.58E-01 | 5.50E-05 | 3.20E-04 | 5.63E-02 | 9.32E-04 | 3.19E-04 | 1.50E-03 | 1.46E-03 | 2.98E-05 | 9.45E-04 |
| **IV** | 5.26E-02 | 1.30E-01 | 4.28E-05 | 1.31E-04 | 4.34E-02 | 7.39E-04 | 2.37E-04 | 1.22E-03 | 1.03E-03 | 1.94E-05 | 8.59E-04 |
| **V** | 6.31E-02 | 1.31E-01 | 5.30E-05 | 5.09E-04 | 2.43E-02 | 9.59E-04 | 2.91E-04 | 2.04E-03 | 2.71E-03 | 6.05E-05 | 3.51E-04 |
| **VI** | 4.38E-02 | 1.46E-01 | 3.88E-05 | 4.74E-04 | 3.54E-02 | 6.11E-04 | 1.68E-04 | 1.48E-03 | 1.65E-03 | 5.37E-05 | 6.39E-04 |
| **VIIa** | 2.38E-02 | 1.89E-01 | 1.88E-05 | 6.38E-05 | 2.21E-02 | 1.90E-03 | 1.03E-04 | 1.11E-03 | 1.75E-03 | 3.16E-05 | 7.38E-03 |
| **VIIb** | 2.76E-02 | 1.39E-01 | 2.24E-05 | 2.23E-04 | 1.81E-02 | 1.13E-03 | 1.37E-04 | 1.35E-03 | 1.15E-03 | 4.39E-05 | 7.56E-05 |
| **VIIIa** | 2.52E-02 | 2.21E-01 | 1.74E-05 | 3.67E-05 | 2.54E-02 | 8.86E-04 | 1.13E-04 | 1.53E-03 | 9.17E-04 | 3.96E-05 | 5.13E-04 |
| **VIIIb** | 2.41E-02 | 1.96E-01 | 2.11E-05 | 6.06E-05 | 2.88E-02 | 8.92E-04 | 1.13E-04 | 1.39E-03 | 6.43E-04 | 2.72E-05 | 6.45E-04 |
| **VIIIc** | 3.87E-02 | 1.79E-01 | 2.43E-05 | 9.23E-05 | 3.08E-02 | 1.57E-03 | 1.47E-04 | 1.89E-03 | 1.01E-03 | 4.26E-05 | 6.99E-04 |
| **IXa** | 3.13E-02 | 2.20E-01 | 1.90E-05 | 4.83E-05 | 2.69E-02 | 6.86E-04 | 1.15E-04 | 9.49E-04 | 8.12E-04 | 3.53E-05 | 3.15E-03 |
| **IXb** | 2.13E-02 | 1.74E-01 | 1.86E-05 | 1.12E-04 | 2.80E-02 | 1.14E-03 | 1.12E-04 | 9.07E-04 | 5.86E-04 | 2.62E-05 | 4.80E-04 |
| **IXc** | 6.21E-02 | 1.85E-01 | 3.17E-05 | 1.55E-04 | 5.71E-02 | 2.09E-03 | 1.55E-04 | 1.29E-03 | 4.75E-04 | 2.86E-05 | 9.00E-04 |
| **X** | 9.28E-02 | 1.94E-01 | 4.15E-05 | 2.39E-04 | 8.47E-02 | 2.74E-03 | 1.92E-04 | 1.70E-03 | 1.07E-03 | 3.46E-05 | 1.75E-03 |
| **XIa** | 4.01E-02 | 1.87E-01 | 2.35E-05 | 8.63E-05 | 4.09E-02 | 1.49E-03 | 1.55E-04 | 1.95E-03 | 7.93E-04 | 3.90E-05 | 1.01E-03 |
| **XIb** | 1.54E-01 | 1.35E-01 | 4.87E-05 | 9.73E-04 | 1.30E-01 | 1.98E-03 | 2.65E-04 | 1.62E-03 | 5.57E-04 | 2.65E-05 | 6.45E-04 |

**Table S5**. Chronic Daily Intake (CDI) for dermal absorption in children

| **Site** | **Al** | **Ti** | **Cr** | **Mn** | **Fe** | **Co** | **Ni** | **Cu** | **Zn** | **Cd** | **Pb** |
| --- | --- | --- | --- | --- | --- | --- | --- | --- | --- | --- | --- |
| **Ia** | 1.12E-01 | 7.05E-02 | 1.85E-03 | 7.54E-04 | 2.52E-01 | 1.85E-03 | 7.90E-04 | 1.33E-02 | 6.73E-03 | 1.52E-04 | 2.41E-03 |
| **Ib** | 1.59E-01 | 1.52E-01 | 1.18E-03 | 9.07E-04 | 2.98E-01 | 1.89E-03 | 7.10E-04 | 8.93E-03 | 5.82E-03 | 2.56E-04 | 2.78E-03 |
| **Ic** | 1.87E-01 | 1.79E-01 | 1.23E-03 | 1.86E-03 | 4.73E-01 | 2.41E-03 | 9.54E-04 | 1.54E-02 | 2.00E-02 | 3.91E-03 | 2.58E-03 |
| **II** | 5.42E-02 | 6.56E-01 | 1.03E-04 | 1.99E-04 | 7.42E-02 | 1.34E-03 | 5.42E-04 | 2.62E-03 | 3.19E-03 | 6.96E-05 | 1.12E-03 |
| **IIIa** | 3.79E-02 | 8.38E-01 | 1.26E-04 | 2.15E-04 | 2.33E-01 | 1.04E-03 | 5.10E-04 | 1.07E-03 | 6.27E-03 | 4.95E-05 | 2.62E-04 |
| **IIIb** | 1.39E-01 | 6.67E-01 | 1.50E-04 | 1.53E-03 | 3.20E-01 | 2.71E-03 | 9.93E-04 | 7.90E-03 | 7.29E-03 | 1.53E-04 | 2.85E-03 |
| **IIIc** | 2.70E-01 | 5.84E-01 | 2.03E-04 | 1.18E-03 | 2.08E-01 | 3.44E-03 | 1.18E-03 | 5.54E-03 | 5.38E-03 | 1.10E-04 | 3.49E-03 |
| **IV** | 1.95E-01 | 4.81E-01 | 1.58E-04 | 4.83E-04 | 1.60E-01 | 2.73E-03 | 8.77E-04 | 4.50E-03 | 3.80E-03 | 7.15E-05 | 3.17E-03 |
| **V** | 2.33E-01 | 4.85E-01 | 1.96E-04 | 1.88E-03 | 9.00E-02 | 3.54E-03 | 1.08E-03 | 7.53E-03 | 1.00E-02 | 2.23E-04 | 1.30E-03 |
| **VI** | 1.62E-01 | 5.39E-01 | 1.44E-04 | 1.75E-03 | 1.31E-01 | 2.26E-03 | 6.21E-04 | 5.47E-03 | 6.10E-03 | 1.98E-04 | 2.36E-03 |
| **VIIa** | 8.81E-02 | 6.99E-01 | 6.96E-05 | 2.36E-04 | 8.16E-02 | 7.02E-03 | 3.79E-04 | 4.09E-03 | 6.47E-03 | 1.17E-04 | 2.73E-02 |
| **VIIb** | 1.02E-01 | 5.12E-01 | 8.28E-05 | 8.24E-04 | 6.68E-02 | 4.18E-03 | 5.08E-04 | 5.00E-03 | 4.27E-03 | 1.62E-04 | 2.79E-04 |
| **VIIIa** | 9.30E-02 | 8.16E-01 | 6.42E-05 | 1.36E-04 | 9.39E-02 | 3.27E-03 | 4.17E-04 | 5.65E-03 | 3.39E-03 | 1.47E-04 | 1.90E-03 |
| **VIIIb** | 8.90E-02 | 7.23E-01 | 7.79E-05 | 2.24E-04 | 1.07E-01 | 3.30E-03 | 4.18E-04 | 5.12E-03 | 2.38E-03 | 1.00E-04 | 2.39E-03 |
| **VIIIc** | 1.43E-01 | 6.60E-01 | 8.97E-05 | 3.41E-04 | 1.14E-01 | 5.79E-03 | 5.44E-04 | 6.97E-03 | 3.72E-03 | 1.57E-04 | 2.58E-03 |
| **IXa** | 1.16E-01 | 8.12E-01 | 7.01E-05 | 1.78E-04 | 9.93E-02 | 2.53E-03 | 4.25E-04 | 3.51E-03 | 3.00E-03 | 1.30E-04 | 1.17E-02 |
| **IXb** | 7.88E-02 | 6.43E-01 | 6.86E-05 | 4.13E-04 | 1.04E-01 | 4.20E-03 | 4.15E-04 | 3.35E-03 | 2.17E-03 | 9.70E-05 | 1.77E-03 |
| **IXc** | 2.30E-01 | 6.84E-01 | 1.17E-04 | 5.74E-04 | 2.11E-01 | 7.72E-03 | 5.71E-04 | 4.79E-03 | 1.76E-03 | 1.06E-04 | 3.33E-03 |
| **X** | 3.43E-01 | 7.18E-01 | 1.53E-04 | 8.85E-04 | 3.13E-01 | 1.01E-02 | 7.11E-04 | 6.28E-03 | 3.97E-03 | 1.28E-04 | 6.46E-03 |
| **XIa** | 1.48E-01 | 6.92E-01 | 8.67E-05 | 3.19E-04 | 1.51E-01 | 5.51E-03 | 5.74E-04 | 7.22E-03 | 2.93E-03 | 1.44E-04 | 3.75E-03 |
| **XIb** | 5.70E-01 | 4.99E-01 | 1.80E-04 | 3.60E-03 | 4.79E-01 | 7.30E-03 | 9.80E-04 | 5.99E-03 | 2.06E-03 | 9.80E-05 | 2.38E-03 |

**Table S6** HQ for males, females, and children

|  | **Males** | | | | | | **Females** | | | | | | **Children** | | | | | |
| --- | --- | --- | --- | --- | --- | --- | --- | --- | --- | --- | --- | --- | --- | --- | --- | --- | --- | --- |
| **Site** | Cd | Cu | Fe | Mn | Pb | Zn | Cd | Cu | Fe | Mn | Pb | Zn | Cd | Cu | Fe | Mn | Pb | Zn |
| **Ia** | 4.62E-02 | 1.01E-01 | 9.58E-02 | 1.64E-03 | 2.08E-01 | 6.83E-03 | 4.11E-02 | 9.00E-02 | 8.52E-02 | 1.46E-03 | 1.85E-01 | 6.07E-03 | 1.52E-01 | 3.33E-01 | 3.15E-01 | 5.39E-03 | 6.84E-01 | 2.24E-02 |
| **Ib** | 7.80E-02 | 6.79E-02 | 1.13E-01 | 1.97E-03 | 2.40E-01 | 5.90E-03 | 6.93E-02 | 6.04E-02 | 1.01E-01 | 1.75E-03 | 2.13E-01 | 5.25E-03 | 2.56E-01 | 2.23E-01 | 3.72E-01 | 6.48E-03 | 7.89E-01 | 1.94E-02 |
| **Ic** | 1.19E+00 | 1.17E-01 | 1.80E-01 | 4.04E-03 | 2.23E-01 | 2.03E-02 | 1.06E+00 | 1.04E-01 | 1.60E-01 | 3.59E-03 | 1.98E-01 | 1.81E-02 | 3.91E+00 | 3.85E-01 | 5.92E-01 | 1.33E-02 | 7.32E-01 | 6.68E-02 |
| **II** | 2.12E-02 | 1.99E-02 | 2.82E-02 | 4.34E-04 | 9.66E-02 | 3.23E-03 | 1.88E-02 | 1.77E-02 | 2.51E-02 | 3.85E-04 | 8.58E-02 | 2.88E-03 | 6.96E-02 | 6.54E-02 | 9.28E-02 | 1.42E-03 | 3.17E-01 | 1.06E-02 |
| **IIIa** | 1.51E-02 | 8.18E-03 | 8.87E-02 | 4.68E-04 | 2.27E-02 | 6.36E-03 | 1.34E-02 | 7.27E-03 | 7.89E-02 | 4.16E-04 | 2.01E-02 | 5.65E-03 | 4.95E-02 | 2.69E-02 | 2.92E-01 | 1.54E-03 | 7.45E-02 | 2.09E-02 |
| **IIIb** | 4.65E-02 | 6.01E-02 | 1.22E-01 | 3.33E-03 | 2.47E-01 | 7.40E-03 | 4.14E-02 | 5.34E-02 | 1.08E-01 | 2.96E-03 | 2.19E-01 | 6.57E-03 | 1.53E-01 | 1.97E-01 | 4.00E-01 | 1.10E-02 | 8.10E-01 | 2.43E-02 |
| **IIIc** | 3.36E-02 | 4.21E-02 | 7.92E-02 | 2.57E-03 | 3.02E-01 | 5.46E-03 | 2.98E-02 | 3.74E-02 | 7.04E-02 | 2.28E-03 | 2.69E-01 | 4.85E-03 | 1.10E-01 | 1.38E-01 | 2.60E-01 | 8.45E-03 | 9.93E-01 | 1.79E-02 |
| **IV** | 2.18E-02 | 3.42E-02 | 6.10E-02 | 1.05E-03 | 2.75E-01 | 3.85E-03 | 1.94E-02 | 3.04E-02 | 5.42E-02 | 9.34E-04 | 2.44E-01 | 3.43E-03 | 7.15E-02 | 1.12E-01 | 2.00E-01 | 3.45E-03 | 9.02E-01 | 1.27E-02 |
| **V** | 6.80E-02 | 5.73E-02 | 3.42E-02 | 4.09E-03 | 1.12E-01 | 1.02E-02 | 6.05E-02 | 5.09E-02 | 3.04E-02 | 3.64E-03 | 9.97E-02 | 9.04E-03 | 2.23E-01 | 1.88E-01 | 1.12E-01 | 1.34E-02 | 3.68E-01 | 3.34E-02 |
| **VI** | 6.04E-02 | 4.16E-02 | 4.98E-02 | 3.81E-03 | 2.04E-01 | 6.18E-03 | 5.37E-02 | 3.70E-02 | 4.43E-02 | 3.39E-03 | 1.81E-01 | 5.50E-03 | 1.98E-01 | 1.37E-01 | 1.64E-01 | 1.25E-02 | 6.71E-01 | 2.03E-02 |
| **VIIa** | 3.55E-02 | 3.11E-02 | 3.11E-02 | 5.12E-04 | 2.36E+00 | 6.56E-03 | 3.16E-02 | 2.77E-02 | 2.76E-02 | 4.55E-04 | 2.10E+00 | 5.83E-03 | 1.17E-01 | 1.02E-01 | 1.02E-01 | 1.68E-03 | 7.75E+00 | 2.16E-02 |
| **VIIb** | 4.94E-02 | 3.80E-02 | 2.54E-02 | 1.79E-03 | 2.42E-02 | 4.33E-03 | 4.39E-02 | 3.38E-02 | 2.26E-02 | 1.59E-03 | 2.15E-02 | 3.85E-03 | 1.62E-01 | 1.25E-01 | 8.35E-02 | 5.89E-03 | 7.93E-02 | 1.42E-02 |
| **VIIIa** | 4.46E-02 | 4.30E-02 | 3.57E-02 | 2.95E-04 | 1.64E-01 | 3.44E-03 | 3.96E-02 | 3.82E-02 | 3.17E-02 | 2.62E-04 | 1.46E-01 | 3.06E-03 | 1.47E-01 | 1.41E-01 | 1.17E-01 | 9.70E-04 | 5.39E-01 | 1.13E-02 |
| **VIIIb** | 3.06E-02 | 3.90E-02 | 4.06E-02 | 4.87E-04 | 2.06E-01 | 2.41E-03 | 2.72E-02 | 3.47E-02 | 3.61E-02 | 4.33E-04 | 1.83E-01 | 2.14E-03 | 1.00E-01 | 1.28E-01 | 1.33E-01 | 1.60E-03 | 6.78E-01 | 7.92E-03 |
| **VIIIc** | 4.79E-02 | 5.31E-02 | 4.33E-02 | 7.41E-04 | 2.23E-01 | 3.78E-03 | 4.26E-02 | 4.72E-02 | 3.85E-02 | 6.59E-04 | 1.99E-01 | 3.36E-03 | 1.57E-01 | 1.74E-01 | 1.42E-01 | 2.44E-03 | 7.34E-01 | 1.24E-02 |
| **IXa** | 3.97E-02 | 2.67E-02 | 3.78E-02 | 3.88E-04 | 1.01E+00 | 3.05E-03 | 3.53E-02 | 2.37E-02 | 3.36E-02 | 3.45E-04 | 8.96E-01 | 2.71E-03 | 1.30E-01 | 8.77E-02 | 1.24E-01 | 1.27E-03 | 3.31E+00 | 1.00E-02 |
| **IXb** | 2.95E-02 | 2.55E-02 | 3.94E-02 | 8.98E-04 | 1.53E-01 | 2.20E-03 | 2.62E-02 | 2.27E-02 | 3.51E-02 | 7.98E-04 | 1.36E-01 | 1.95E-03 | 9.70E-02 | 8.38E-02 | 1.30E-01 | 2.95E-03 | 5.04E-01 | 7.22E-03 |
| **IXc** | 3.22E-02 | 3.64E-02 | 8.02E-02 | 1.25E-03 | 2.88E-01 | 1.78E-03 | 2.86E-02 | 3.24E-02 | 7.13E-02 | 1.11E-03 | 2.56E-01 | 1.58E-03 | 1.06E-01 | 1.20E-01 | 2.64E-01 | 4.10E-03 | 9.45E-01 | 5.85E-03 |
| **X** | 3.89E-02 | 4.78E-02 | 1.19E-01 | 1.92E-03 | 5.58E-01 | 4.03E-03 | 3.46E-02 | 4.25E-02 | 1.06E-01 | 1.71E-03 | 4.96E-01 | 3.58E-03 | 1.28E-01 | 1.57E-01 | 3.91E-01 | 6.32E-03 | 1.83E+00 | 1.32E-02 |
| **XIa** | 4.38E-02 | 5.50E-02 | 5.75E-02 | 6.94E-04 | 3.24E-01 | 2.97E-03 | 3.90E-02 | 4.89E-02 | 5.11E-02 | 6.16E-04 | 2.88E-01 | 2.64E-03 | 1.44E-01 | 1.81E-01 | 1.89E-01 | 2.28E-03 | 1.07E+00 | 9.77E-03 |
| **XIb** | 2.98E-02 | 4.56E-02 | 1.82E-01 | 7.82E-03 | 2.06E-01 | 2.09E-03 | 2.65E-02 | 4.05E-02 | 1.62E-01 | 6.95E-03 | 1.83E-01 | 1.86E-03 | 9.80E-02 | 1.50E-01 | 5.98E-01 | 2.57E-02 | 6.77E-01 | 6.86E-03 |

**Table S7**. Comparison of the average concentration of the studied PHEs with shale rocks and SQGs

| **Metals** | **Shale** | **This study (average concentrations)** | **SQG** | | | **TEL** | **PEL** | **ERL** | **ERM** |
| --- | --- | --- | --- | --- | --- | --- | --- | --- | --- |
|  |  |  | ***Non-polluted*** | ***Moderate polluted*** | ***Heavily polluted*** |  |  |  |  |
| Cu | 45 | 12.66 | < 25 | 25-50 | >50 | 18.70 | 110 | 34 | 270 |
| Cd | 0.30 | 125.95 | - | - | - | 0.68 | 4.20 | 1.20 | 9.60 |
| Zn | 95 | 10.76 | < 90 | 90-200 | >200 | 124 | 270 | 150 | 410 |
| Ni | 68 | 1.38 | < 20 | 20-50 | >50 | 15.90 | 43 | 20.90 | 51.60 |

**Table S8** Correlation matrix of the investigated elements in sediment samples

|  | **Li** | **B** | **Na** | **Mg** | **Al** | **K** | **Ca** | **Ti** | **Cr** | **Mn** | **Fe** | **Co** | **Ni** | **Cu** | **Zn** | **Ga** | **Se** | **Sr** | **Ag** | **Cd** | **In** | **Ba** | **Pb** |
| --- | --- | --- | --- | --- | --- | --- | --- | --- | --- | --- | --- | --- | --- | --- | --- | --- | --- | --- | --- | --- | --- | --- | --- |
| Li | 1 |  |  |  |  |  |  |  |  |  |  |  |  |  |  |  |  |  |  |  |  |  |  |
| B | -.334 |  |  |  |  |  |  |  |  |  |  |  |  |  |  |  |  |  |  |  |  |  |  |
| Na | .469^*^ | .012 |  |  |  |  |  |  |  |  |  |  |  |  |  |  |  |  |  |  |  |  |  |
| Mg | .101 | .343 | .088 |  |  |  |  |  |  |  |  |  |  |  |  |  |  |  |  |  |  |  |  |
| Al | **.961^**^** | -.299 | .413 | -.021 |  |  |  |  |  |  |  |  |  |  |  |  |  |  |  |  |  |  |  |
| K | .454^*^ | .107 | .560^**^ | .201 | .472^*^ |  |  |  |  |  |  |  |  |  |  |  |  |  |  |  |  |  |  |
| Ca | -.551^**^ | -.015 | -.164 | -.146 | **-.606^**^** | -.350 |  |  |  |  |  |  |  |  |  |  |  |  |  |  |  |  |  |
| Ti | -.186 | -.495^*^ | -.316 | -.499^*^ | -.170 | -.153 | .209 |  |  |  |  |  |  |  |  |  |  |  |  |  |  |  |  |
| Cr | -.038 | .464^*^ | .232 | .407 | -.020 | .013 | .168 | **-.882^**^** |  |  |  |  |  |  |  |  |  |  |  |  |  |  |  |
| Mn | **.833^**^** | -.032 | .213 | .315 | **.789^**^** | .313 | -.511^*^ | -.409 | .169 |  |  |  |  |  |  |  |  |  |  |  |  |  |  |
| Fe | **.682^**^** | -.055 | .371 | .215 | **.628^**^** | .070 | -.045 | -.458^*^ | .478^*^ | **.673^**^** |  |  |  |  |  |  |  |  |  |  |  |  |  |
| Co | .512^*^ | -.421 | .344 | -.209 | .564^**^ | .427 | -.285 | .276 | -.332 | .147 | .145 |  |  |  |  |  |  |  |  |  |  |  |  |
| Ni | **.641^**^** | .239 | .359 | .421 | .598^**^ | .543^*^ | -.408 | -.488^*^ | .308 | **.702^**^** | .580^**^ | -.063 |  |  |  |  |  |  |  |  |  |  |  |
| Cu | .226 | .284 | .431 | .431 | .170 | .374 | .151 | -.787^**^ | .814^**^ | .373 | .555^**^ | -.092 | .480^*^ |  |  |  |  |  |  |  |  |  |  |
| Zn | .081 | .427 | .195 | .415 | -.042 | .141 | .388 | -.530^*^ | .527^*^ | .333 | .440^*^ | -.305 | .453^*^ | **.692^**^** |  |  |  |  |  |  |  |  |  |
| Ga | **.900^**^** | -.367 | .462^*^ | -.005 | **.911^**^** | .381 | -.384 | -.182 | .065 | **.645^**^** | **.726^**^** | **.606^**^** | .510^*^ | .258 | .082 |  |  |  |  |  |  |  |  |
| Se | -.171 | -.220 | -.080 | -.204 | -.125 | -.142 | .300 | .232 | -.072 | -.212 | -.183 | -.147 | -.236 | -.152 | -.137 | -.172 |  |  |  |  |  |  |  |
| Sr | -.539^*^ | -.285 | -.360 | **-.670^**^** | -.457^*^ | -.396 | .478^*^ | **.700^**^** | -.431 | **-.672^**^** | -.489^*^ | .048 | -.737^**^ | -.554^**^ | -.446^*^ | -.437^*^ | .354 |  |  |  |  |  |  |
| Ag | .218 | -.245 | .057 | -.043 | .303 | .214 | -.011 | .210 | -.123 | -.035 | .180 | .592^**^ | .023 | -.059 | -.081 | .528^*^ | -.024 | .088 |  |  |  |  |  |
| Cd | .193 | .064 | .309 | .188 | .037 | .010 | .464^*^ | -.454^*^ | .458^*^ | .278 | .516^*^ | -.158 | .270 | **.668^**^** | **.865^**^** | .242 | -.063 | -.348 | -.081 |  |  |  |  |
| In | .318 | -.236 | .261 | .010 | .384 | .310 | -.051 | .049 | .019 | .057 | .308 | **.654^**^** | .067 | .138 | -.016 | **.615^**^** | -.106 | -.001 | **.955^**^** | .020 |  |  |  |
| Ba | -.120 | .204 | .216 | .160 | -.148 | -.030 | -.034 | -.054 | .018 | -.158 | -.078 | -.282 | .157 | -.183 | .016 | -.180 | .335 | -.225 | -.206 | -.055 | -.289 |  |  |
| Pb | -.178 | .194 | -.097 | -.195 | -.078 | .034 | .179 | .210 | -.132 | -.214 | -.175 | .355 | -.285 | -.148 | -.001 | -.071 | .289 | .165 | .202 | -.064 | .126 | .193 |  |
| Bi | .188 | -.154 | .289 | -.121 | .289 | .359 | .013 | .199 | -.105 | -.093 | .112 | **.681^**^** | -.016 | -.022 | -.059 | .472^*^ | .072 | .108 | **.900^**^** | -.073 | **.895^**^** | -.033 | .445^*^ |

*. Correlation is significant at the 0.05 level (1-tailed). **. Correlation is significant at the 0.01 level (2-tailed).

**Table S9** Component matrix in marine sediments

|  | **Component Matrix^a^** | | |
| --- | --- | --- | --- |
|  | 1 | 2 | 3 |
| **Li** | 0.819 | 0.385 | -0.317 |
| **B** | 0.066 | -0.602 | 0.129 |
| **Na** | 0.574 | 0.088 | 0.128 |
| **Mg** | 0.419 | -0.445 | -0.014 |
| **Al** | 0.773 | 0.482 | -0.300 |
| **K** | 0.548 | 0.226 | -0.005 |
| **Ca** | -0.396 | -0.277 | 0.710 |
| **Ti** | -0.623 | 0.610 | -0.079 |
| **Cr** | 0.448 | -0.620 | 0.368 |
| **Mn** | 0.801 | -0.009 | -0.375 |
| **Fe** | 0.789 | -0.041 | 0.124 |
| **Co** | 0.290 | 0.829 | 0.136 |
| **Ni** | 0.801 | -0.182 | -0.236 |
| **Cu** | 0.671 | -0.479 | 0.390 |
| **Zn** | 0.474 | -0.581 | 0.455 |
| **Ga** | 0.798 | 0.514 | 0.005 |
| **Se** | -0.320 | 0.062 | 0.165 |
| **Sr** | -0.790 | 0.331 | 0.223 |
| **Ag** | 0.225 | 0.691 | 0.504 |
| **Cd** | 0.456 | -0.433 | 0.472 |
| **In** | 0.381 | 0.649 | 0.533 |
| **Ba** | -0.083 | -0.225 | -0.105 |
| **Pb** | -0.196 | 0.240 | 0.406 |
| **Bi** | 0.213 | 0.692 | 0.581 |
| **Total** | 7.35 | 5.17 | 2.81 |
| **% of variance** | 30.61 | 21.56 | 11.72 |
| **% of cumulative** | 30.61 | 52.17 | 63.88 |

a: 3 components extracted.


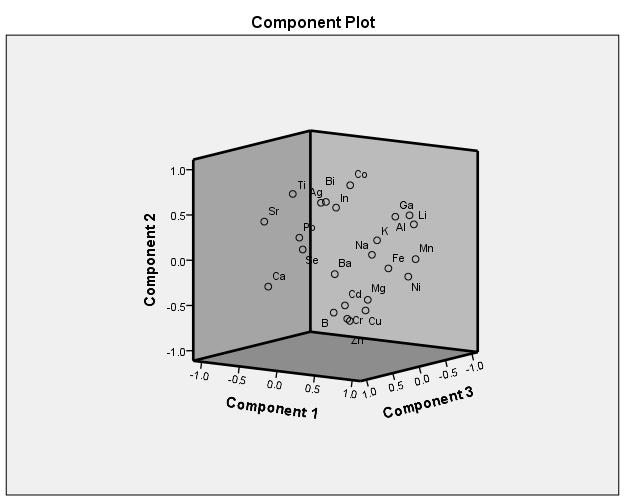


**Figure S1** Principal Component analysis plot.

**Table S10** Comparison between HMs concentrations in present study with those obtained elsewhere in the Mediterranean Sea

| **Location** | **Cd** | **Cr** | **Cu** | **Co** | **Mn** | **Ni** | **Pb** | **Zn** | **Reference** |
| --- | --- | --- | --- | --- | --- | --- | --- | --- | --- |
| **Western Mediterranean Sea Egypt** | 0.101-7.983 | 0.131-3.766 | 2.193-31.417 | 2.12- 20.662 | 0.277-7.343 | 0.774-2.408 | 0.535-55.644 | 3.584-40.894 | Present Study |
| **Mediterranean Sea, Egypt** | 0.04–0.47 | 4.08–297.95 | 0.46–26.26 | 0.43–26.39 | 17–1086 | 1.65–60.25 | 3.34–53.67 | 2.05–62.21 | (Soliman et al) |
| **Malaga bay, (Spain) Mediterranean Sea** | 0.021–0.283 | 4.31–26 | 6.57–21.2 | - | - | 9.48–40.2 | 7.92–37.1 | - | (Castillo et al.) |
| **Mediterranean Sea, Morocco** | 0.14–0.27 | 88.40–160.97 | 4.09–29.12 | 18.06–31.7 | 256.56–651.66 | 3.19–79.89 | 33.11–47.97 | 64.82–110.77 | (Omar et al.) |
| **Mediterranean Sea, Turkey** | - | - | - | - | 283–1192 | 28–240 | 91.3–751 | 86–970 | (Neşer et al) |
| **Ivra Complex Italy** | - | 2568–2984 | - | 100–117 | 968–1053 | 2040–2438 | 0.25 | - | (Hartmann, and Wedepohl,) |
| **Mediterranean Sea, Libya** | 5–10.5 | 14.8–24.9 | 9.1–22.7 | 8.2–18.1 | 14.3–49.4 | 11.6–29.9 | 8.9–56.9 | 11.6–30.5 | (Soliman et al) |
| **Eastern Mediterranean Sea, Egypt** | 1.8–2.3 | - | 4–9.4 | - | 200.8–254.3 | - | 18.4–24.8 | 33.1–42.2 | (El-Serehy et al.) |
| **Average shale** | 0.3 | 90 | 45 | 19 | 850 | 68 | 20 | 95 | (Turekian et al.) |

**References**

Soliman, N.F., Nasr, S.M. and Okbah, M.A. Potential ecological risk of heavy metals in sediments from the Mediterranean coast, Egypt. *Journal of Environmental Health Science and Engineering*, *13*, 1-12 (2015).

Castillo, M.A., Trujillo, I.S., Alonso, E.V., de Torres, A.G. and Pavón, J.C. Bioavailability of heavy metals in water and sediments from a typical Mediterranean Bay (Málaga Bay, Region of Andalucía, Southern Spain). *Marine pollution bulletin*, *76*(1-2), 427-434 (2013).

Omar, M.B., Mendiguchía, C., Er-Raioui, H., Marhraoui, M., Lafraoui, G., Oulad-Abdellah, M.K., García-Vargas, M. and Moreno, C. Distribution of heavy metals in marine sediments of Tetouan coast (North of Morocco): natural and anthropogenic sources. *Environmental Earth Sciences*, *74*, 4171-4185 (2015).

Neşer, G., Kontas, A., Ünsalan, D., Altay, O., Darılmaz, E., Uluturhan, E., Küçüksezgin, F., Tekoğul, N. and Yercan, F. Polycyclic aromatic and aliphatic hydrocarbons pollution at the coast of Aliağa (Turkey) ship recycling zone. *Marine Pollution Bulletin*, *64*(5), 1055-1059 (2012).

Hartmann, G. and Wedepohl, K.H. The Composition of Peridotite Tectonites from the Ivrea Complex, Northern Italy: Residues from Melt Extraction. Geochimica et Cosmochimica Acta, 57, 1761-1782 (1993). <http://dx.doi.org/10.1016/0016-7037(93)90112-A>

El-Serehy, H.A., Aboulela, H., Al-Misned, F., Kaiser, M., Al-Rasheid, K. and El-Din, H.E. Heavy metals contamination of a Mediterranean coastal ecosystem, Eastern Nile Delta, Egypt. *Turkish Journal of Fisheries and Aquatic Sciences*, *12*(4), (2012).

Turekian, K.K. and Wedepohl, K.H. Distribution of the elements in some major units of the earth's crust. *Geological society of America bulletin*, *72*(2), 175-192 (1961).
